# Stroke in non-bacterial thrombotic endocarditis: a patient-level systematic review of clinical characteristics, management, and outcomes

**DOI:** 10.3389/fstro.2026.1915602

**Published:** 2026-08-14

**Authors:** Ahmed Aljabali, Deema Alhayali, Hallie Taylor-Wedge, Todd A. Laffaye, Majd Al-Ahmed, Ayham Harahsheh, Mohammed Alamoush, Khaled Dweik, Mayowa A. Osundiji, Amir A. Mbonde, Cumara B. O'Carroll, Bart M. Demaerschalk, Ehab Harahsheh

**Affiliations:** 1 School of Medicine, Jordan University of Science and Technology, Irbid, Jordan; 2 School of Medicine, University of Sharjah, Sharjah, United Arab Emirates; 3 Department of Neurology, Mayo Clinic College of Medicine and Science, Phoenix, AZ, United States; 4 Mayo Clinic Alix School of Medicine, Mayo Clinic Arizona, Scottsdale, AZ, United States; 5 Department of Internal Medicine, University of Missouri, Columbia, MO, United States; 6 Department of Internal Medicine, Islamic Hospital, Amman, Jordan; 7 School of Medicine, University of Jordan, Amman, Jordan; 8 Department of Clinical Genomics, Mayo Clinic Arizona, Phoenix, AZ, United States; 9 Department of Neurology, Medical College of Georgia, Augusta, GA, United States; 10 Department of Neurology, University of Arizona, Tucson, AZ, United States

**Keywords:** non-bacterial thrombotic endocarditis, stroke, recurrent stroke, anticoagulation, systematic review

## Abstract

**Background and Purpose:**

Stroke is a frequent manifestation of non-bacterial thrombotic endocarditis (NBTE), yet current knowledge largely stems from case reports and small case series. This systematic review summarizes stroke presentation, imaging features, management, and outcomes in patients with NBTE-related stroke.

**Methods:**

We conducted a patient-level systematic review of published case reports and case series. PubMed, MEDLINE, Embase, Web of Science, and PsycINFO were searched from inception through August 2025 for studies reporting individual patients with NBTE-related stroke. Extracted data included demographics, vascular risk factors, stroke presentation, imaging features, NBTE etiology, treatment (unfractionated heparin/low-molecular-weight heparin [UFH/LMWH], vitamin K antagonists [VKA], direct oral anticoagulants [DOACs], and/or valvular surgery), and outcomes. Multivariable regression was used to explore factors associated with recurrent stroke and mortality.

**Results:**

We included 236 patients with NBTE and stroke from 218 studies (median age 56 years [IQR: 43–63], 63% female). Left hemispheric stroke syndromes predominated among clinical presentations (27%), and multi-vessel territory ischemic infarcts (>2 territories) were observed in 65%. Conventional vascular risk factors were infrequent (0%−21%), whereas deep vein thrombosis (43%) and systemic embolism (41%) frequently preceded or coincided with stroke onset. Malignancy (70%) and autoimmune disease (25%) were the main NBTE etiologies. Anticoagulation was initiated in 70%, and 21% underwent valvular surgery. Over a median follow-up of 2.0 months, stroke recurred in 43% and death occurred in 49% of patients. Malignant etiology (aOR 10.3, *P* < 0.001) and metastases (aOR 3.9, *P* < 0.001) were associated with increased mortality, while VKA use (aOR 0.08, *P* < 0.001) and autoimmune etiology (aOR 0.1, *P* < 0.001) were associated with lower mortality. Malignancy (aOR 2.8, *P* < 0.001) and DOAC use (aOR 5.2, *P* = 0.012) were associated with increased risk of recurrent stroke.

**Conclusion:**

NBTE should be considered in stroke patients lacking conventional vascular risk factors, particularly those with malignancy or autoimmune disease. Malignancy and DOAC use were associated with higher recurrent stroke risk, while VKA use was associated with lower mortality; however, these anticoagulant-class findings should be considered hypothesis-generating and interpreted cautiously given the case-report evidence base, small treatment subgroups, potential confounding, and limited follow-up.

**What this study adds:**

By synthesizing patient-level data from published case reports and case series, this review provides a stroke-focused characterization of NBTE, including clinical presentation, infarct topography, underlying etiologies, management strategies, recurrent cerebrovascular events, and mortality. The findings highlight multifocal infarction, malignancy-associated NBTE, and recurrent stroke despite anticoagulation as key clinical concerns, while emphasizing that observed associations with anticoagulant class should be viewed as hypothesis-generating rather than definitive treatment guidance

## Introduction

Non-bacterial thrombotic endocarditis (NBTE), commonly referred to as marantic endocarditis, is a form of non-infective endocarditis characterized by sterile vegetations on cardiac valves that are predominantly composed of fibrin and platelets. (Lopez et al., 1987; El-Shami et al., 2007). Although NBTE has been described in association with several conditions, it is most frequently observed in patients with systemic malignancies, often at advanced stages, and has been associated with worse outcomes and reduced overall survival in this population (Zmaili et al., 2021). NBTE can lead to systemic embolism, and ischemic cerebrovascular events are among the most common clinical manifestations of embolization (Alhuarrat et al., 2024). Despite this, the literature on stroke and cerebrovascular events in NBTE is largely limited to case reports, case series, postmortem studies, and a small number of observational studies (Steiner, 1993; Eiken et al., 2001; Oakley et al., 2025; Singhal et al., 2002). Thus, we conducted this systematic review of published case reports and case series to describe patient-level clinical presentations, neuroimaging features, management strategies, and outcomes among patients with NBTE-related stroke.

## Methods

### Literature search methodology

A comprehensive literature search was performed in PubMed, MEDLINE, Embase, Web of Science, and PsycINFO from inception to August 1, 2025. Search terms combined controlled vocabulary and free-text terms, including “non-bacterial thrombotic endocarditis,” “marantic endocarditis,” “non-infective endocarditis,” “stroke,” “ischemic stroke,” “cerebral infarction,” “hemorrhagic stroke,” “cerebral hemorrhage,” and “brain bleed.” Boolean operators (“AND,” “OR”) were applied. Reference lists of included articles were manually screened to identify additional eligible studies. The literature search was performed by the last author (E.H.). The search methodology is outlined in the Supplementary Data.

The main inclusion criteria in this review were case reports and case series articles describing NBTE-related stroke patients. NBTE-related stroke, in this review, was defined as any acute stroke syndrome that occurred **within 30 days** of the diagnosis of NBTE. Review articles, cohort studies, articles describing NBTE patients without stroke, case series and case reports without sufficient details pertaining to the clinical presentation, imaging features, management, and outcomes of patients with NBTE-related stroke were excluded. In addition, relevant case reports and case series published in languages other than English were included if authors were able to obtain a high-quality translation for the medical content and sufficient details as described above were provided.

The protocol was not prospectively registered in PROSPERO. To improve transparency and reproducibility, the study question, eligibility criteria, outcomes of interest, and data extraction fields were predefined before full-text review, and reporting adhered closely to Preferred Reporting Items for Systematic Reviews and Meta-Analyses (PRISMA) recommendations.

### Screening process

All retrieved articles were imported into Covidence, a web-based collaboration platform designed to streamline systematic and other literature reviews. Two authors (E.H. and M.A.) independently screened titles and abstracts, followed by full-text assessment based on predefined inclusion and exclusion criteria. Irrelevant studies and duplicate reports involving the same patients were excluded. Disagreements were resolved by consensus.

### Data extraction

Two authors (A.A. and H.T.) systematically extracted relevant data from the included articles using a standardized Excel spreadsheet. A third author (E.H.) independently reviewed all included materials to verify the accuracy and completeness of extracted data. Discrepancies were resolved by consensus. The following information was collected for each patient with NBTE-related stroke: first author's name and country of publication, year of publication, number of included patients, demographic data, vascular risk factors, clinical stroke presentation, neuroimaging distribution of infarcts, systemic embolization, method of NBTE diagnosis and vegetation detection, cardiac valves involved, vegetation size and mobility when reported, NBTE etiology, anticoagulation status before NBTE diagnosis and stroke presentation, NBTE management, cancer-directed therapy for malignancy-associated NBTE, immunomodulatory treatment for autoimmune-associated NBTE, and time from NBTE diagnosis to last follow-up or death.

Data on recurrent ischemic or hemorrhagic cerebrovascular events, ischemic stroke recurrence despite anticoagulation, anticoagulation-related bleeding including intracerebral hemorrhage, and mortality including in-hospital death were also extracted. Clinical stroke syndromes and neuroimaging distribution of infarcts were adjudicated by E.H., a vascular neurologist, based on independent review of available images and reported findings in the included articles.

### Quality assessment

Each included case report and case series was assessed by E.H. for quality and risk of bias across four domains: selection, ascertainment, causality, and reporting. This evaluation used the methodological assessment tool proposed by Murad et al. (2018) for case reports and case series, with a maximum score of 6, as questions 5 and 6 from the original tool were deemed not applicable to this review (Supplementary Table 1). Studies scoring 1–2 were categorized as high risk of bias, those scoring 3–4 as intermediate risk, and those scoring 5–6 as low risk of bias.

### Statistical analysis

Descriptive statistics were used to summarize patient-level data extracted from the included case reports and case series. Categorical variables were reported as frequencies and percentages, while continuous variables were presented as means with standard deviations (SD) or medians with interquartile ranges (IQR), as appropriate. Effect sizes were estimated by calculating odds ratios (ORs) with corresponding 95% confidence intervals (CIs).

A univariate analysis was initially performed, followed by multivariable logistic regression models adjusted for age, sex, malignant and autoimmune etiologies, post-NBTE/stroke anticoagulation, and valvular intervention. These exploratory models were used to estimate adjusted ORs for all-cause mortality, recurrent ischemic stroke, and recurrent stroke despite anticoagulation. Given the retrospective synthesis of published case reports and case series, all regression findings were interpreted as associations rather than causal effects. Prior to conducting multivariable regression analysis, collinearity diagnostics were performed to evaluate potential multicollinearity among independent variables. Variance inflation factors (VIFs) and tolerance values were calculated for all candidate predictors. Variables demonstrating significant multicollinearity (e.g., VIF >5 or tolerance < 0.2) were carefully assessed, and where appropriate, correlated variables were removed or not entered simultaneously into the final models. Statistical significance was defined as a two-tailed *p*-value < 0.05. All analyses were conducted using Jamovi software (version 2.4.5, Sydney, AUS).

### Standard protocol approvals, registrations, and patient consents

Institutional review board approval and informed consent were not required because this systematic review used previously published data.

## Results

### Study identification and selection

**Figure 1:**
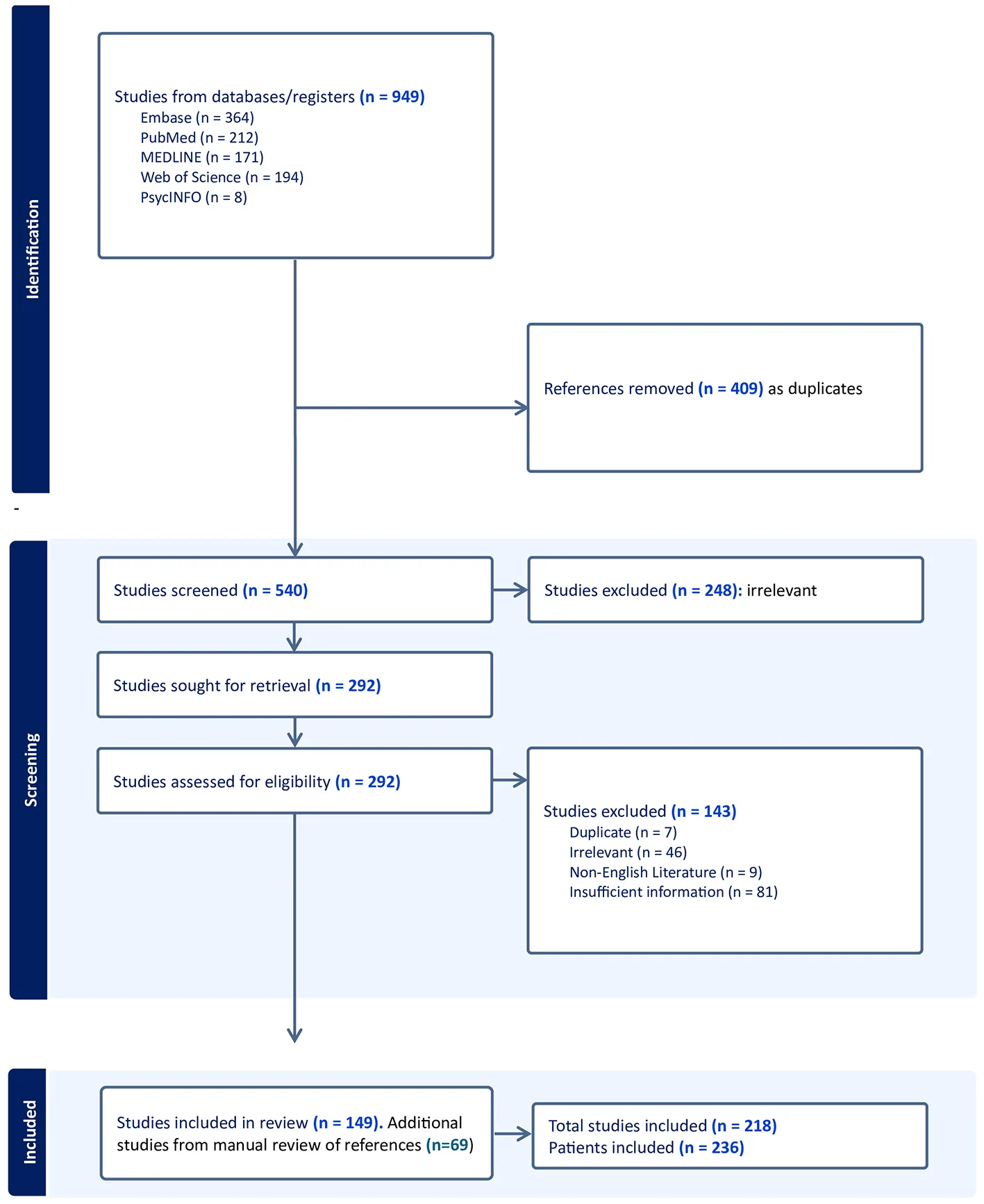
PRISMA flow diagram for study identification, screening, eligibility assessment, and inclusion. PRISMA: preferred reporting items for systematic reviews and meta-analyses.

Using the search strategy outlined above and detailed in the Supplementary Data, 949 studies were identified: 212 from PubMed, 364 from Embase, 171 from MEDLINE, 194 from Web of Science, and 8 from PsycINFO (Figure 1). After removal of 409 duplicate records, 540 articles remained for title and abstract screening. Of these, 248 were excluded as not relevant to the review topic, and 292 full-text articles were assessed for eligibility. Among these, 143 were excluded mainly because they were irrelevant or lacked sufficient information on patient presentation, management, or outcomes. Ultimately, 149 studies published from 1981 to 2025, comprising 7 case series and 142 case reports, met the inclusion criteria. Manual review of reference lists identified an additional 69 eligible studies. These 218 studies provided adequate clinical details for 236 patients who presented with stroke within 1 month of NBTE diagnosis. Approximately 30% of included studies were assessed as low risk of bias, 61% as intermediate risk, and 9% as high risk.

### Baseline characteristics

A total of 236 patients with NBTE-associated stroke were identified, with a median age of 56 years (IQR, 43–63), and 63% were female (Table 1). Most patients had few or no traditional vascular risk factors. Notably, 43% had a prior history of thrombotic events, including DVT and/or PE, most of which occurred within days to weeks before or after NBTE diagnosis.

**Table 1:** Demographics, NBTE etiology and valvular abnormalities.

Variable	Number (%)
**Age, median (IQR)**	56 (43-63)
**Gender**	
Male	88 (37%)
Female	148 (63%)
**Vascular risk factors**	
History of stroke/TIA	34 (14%)
Hypertension	49 (21%)
Dyslipidemia	20 (8%)
Diabetes Mellitus	18 (8%)
Coronary artery disease	7 (3%)
Carotid artery stenosis	2 (0.8%)
Atrial fibrillation	9 (4%)
Pulmonary embolism/DVT	101 (43%)
Obstructive sleep apnea	0 (0)
Smoking history	32 (14%)
**NBTE etiology**	
Malignancy	166 (70%)
Presence of metastases	97 (58%)
Autoimmune^*^	58 (25%)
Others	6 (2%)
Idiopathic	9 (4%)
**Valvular abnormalities**	
Abnormal valve	236 (100%)
Mitral	160 (68%)
Aortic	98 (42%)
Tricuspid	12 (5%)
Pulmonary	4 (2%)
Two concomitant valves	36 (15%)
Three concomitant valves	2 (0.8%)
Four concomitant valves	2 (0.8%)
**Vegetations**	
Vegetations size and mobility report	132 (56%), 100 (42%)
>1 cm	60 (45%)
≤ 1 cm	72 (55%)
Mobile	73 (73%)
**Method of diagnosis** ^**^	
TTE only	44 (19%)
TEE only	116 (49%)
TTE+TEE	49 (21%)
Autopsy/biopsy	24 (10%) ^¶^
Cardiac MRI/CT	3 (1%)

^*^Three patients had concomitant autoimmune and malignancy etiologies and were counted in more than one category, thus the overall percentage adds to >100%.

^**^two patients the methods of diagnosis were not specified.

^¶^One patient was diagnosed with premortem TTE and then confirmed with an autopsy, and one patient was diagnosed based on biopsy.

Abbreviations: CT: computed tomography; DVT: deep venous thrombosis; MRI: magnetic resonance imaging; NBTE: non-bacterial thrombotic endocarditis; TEE: transesophageal echocardiogram; TTE: transthoracic echocardiogram; TIA: transient ischemic attack.

### NBTE diagnosis and etiology

All included patients had a confirmed diagnosis of NBTE either during life (*n* = 215; 91%) or postmortem (*n* = 21; 9%). Premortem diagnosis was established via TEE in 70% (*n* = 165) of cases either alone (*n*=116, 49%) or in combination with TTE (*n* = 49, 21%). TTE alone was sufficient to establish the diagnosis in only 19% (*n* = 44) of cases. The mitral valve was the most frequently involved cardiac valve (*n* = 160, 68%), followed by the aortic valve (*n* = 98, 42%). Concomitant involvement of two or more valves was observed in 15% (*n* = 36) of patients. Among 132 and 100 patients who had their vegetation size and mobility reported, 45% (*n* = 65) had vegetations measuring ≥1 cm in size, and 73% (*n* = 73) had mobile vegetations, respectively.

**Figure 2:**
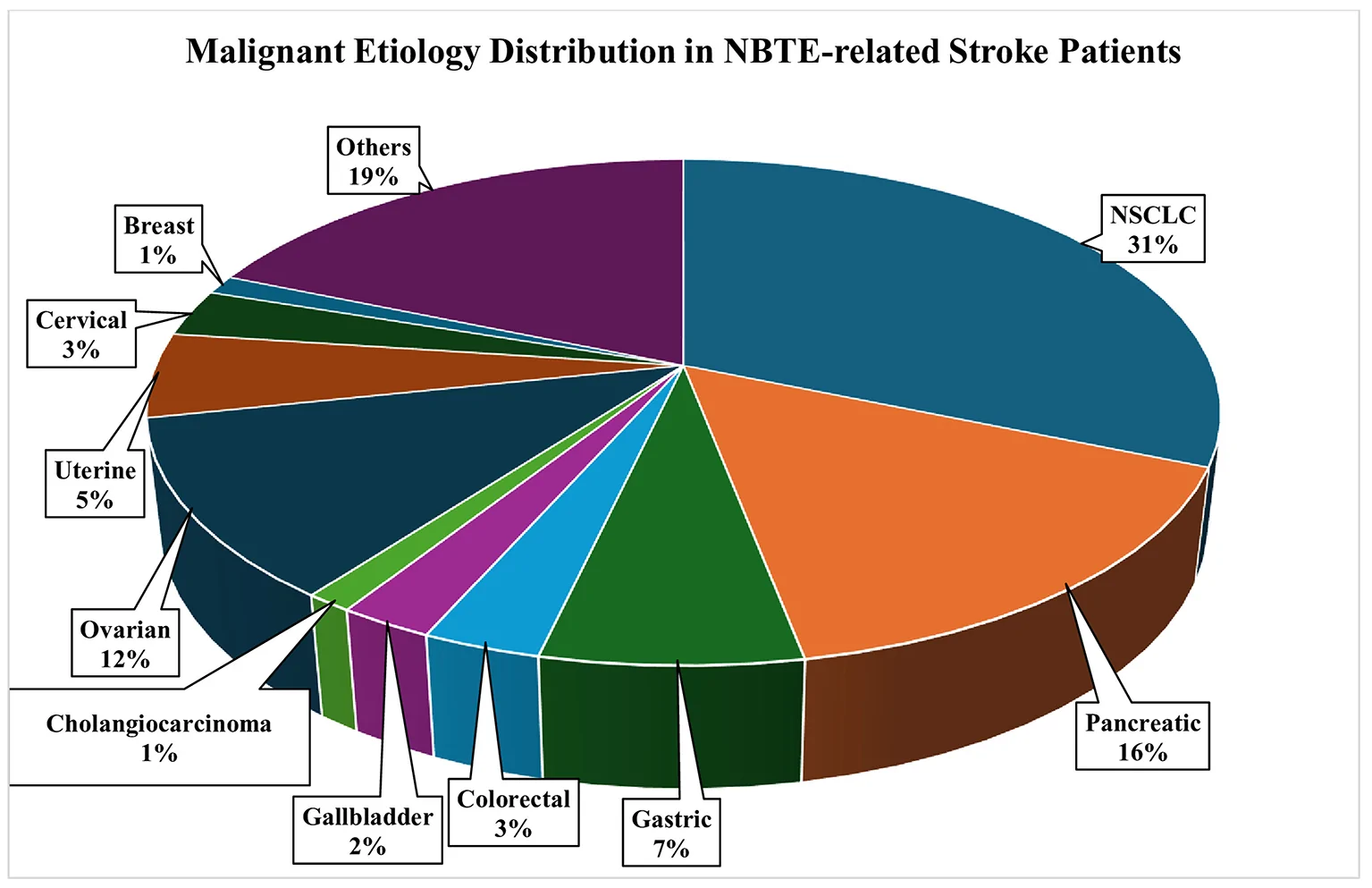
Distribution of malignancy types associated with NBTE in this systematic review. NBTE: non-bacterial thrombotic endocarditis; NSCLC: non-small cell lung cancer.

Systemic malignancies accounted for the majority (*n* = 166, 70%) of NBTE cases, with 58% (*n* = 97) of these being metastatic at the time of NBTE diagnosis, followed by autoimmune disorders representing 25% (*n* = 58). The most frequently observed cancers were non-small cell lung cancer (31%, *n* = 52), gastrointestinal (31%, *n* = 52), and cancers of breast and reproductive organs (22%, *n* = 36) (Figure 2). Among autoimmune causes, antiphospholipid syndrome was the most prevalent (81%, *n* = 47). Only 4% (*n* = 9) of cases had no identifiable cause despite thorough investigations for malignancy and autoimmune diseases.

### Clinical stroke syndromes and neuroimaging features

Approximately 92% (*n* = 218) of patients included in this review presented with clinical stroke syndromes, while the remaining 8% (*n* = 18) were asymptomatic neurologically but showed evidence of acute ischemic infarcts on neuroimaging (Table 2). Neuroimaging revealed acute or subacute ischemic infarcts in 94% (*n* = 223) of cases, with only 0.4% (*n* = 1) demonstrating hemorrhagic infarcts (*n* = 1), and the remaining 5% (*n* = 12) exhibiting a combination of both. About 65% (*n* = 154) of patients had ischemic infarcts affecting multiple vessel territories (>2). Conversely, 18% (*n* = 43) of patients had ischemic infarcts confined to a single vessel territory. Systemic imaging and/or autopsy identified additional systemic emboli and ischemic infarcts in other organs in 41% (*n* = 97) of patients, most commonly affecting the spleen (26%, *n* = 61) and kidneys (26%, *n* = 62), with involvement of two or more organs observed in 28% (*n* = 67) of cases.

**Table 2:** Storke clinical presentation, neuroimaging features, management, and outcomes.

Variable	Number (%)
**Clinical presentation**	
Clinical stroke syndrome	218 (92%)
Right hemispheric stroke syndrome	50 (23%)
Left hemispheric stroke syndrome	59 (27%)
Posterior circulation stroke syndrome	45 (20%)
>1 Stroke syndrome	57 (26%)
Unspecified	10 (4%)
Asymptomatic with neuroimaging evidence of ischemic infarcts	18 (8%)
Systemic embolism	97 (41%)
Spleen	61 (26%)
Kidney	62 (26%)
Heart	20 (8%)
Lower limb ischemia	25 (11%)
>=2 sites of systemic embolism	67 (28%)
**Neuroimaging**	
Ischemic vs hemorrhagic stroke	
Ischemic stroke	223 (94.6%)
Hemorrhagic stroke	1 (0.4%)
Ischemic and hemorrhagic stroke	12 (5%)
Vessel territory	
Single vessel territory	43 (19%)
Two vessel territory	34 (15%)
Multivessel territory	154 (66%)
**Anticoagulation**	
Anticoagulation prior to stroke diagnosis	53 (22%)
DOAC	38 (72%)
VKA	3 (6%)
LMWH	9 (17%)
Unknown AC	3 (5%)
Anticoagulation after stroke diagnosis	165 (70%)
UFH/LWMH	87 (53%)
VKA	27 (16%)
DOAC	18 (11%)
UFH/LMWH followed by VKA	33 (20%)
**Recurrent ischemic stroke**	101 (43%)
Recurrent ischemic stroke despite AC	59 (59%)
LMWH/UFH	35/120 (29%)
VKA	11/60 (18%)
DOAC	10/18 (56%)
**Bleeding events following AC**	34 (14%)
ICH	21 (6%)
**Death related to NBTE or stroke**	115 (49%)
Inpatient mortality	95 (83%)
**Median time stroke diagnosis to death, months (IQR)**	(0.35-2)
**Median time stroke diagnosis to last follow-up, months (IQR)**	2.0 (0.5-5)
Malignancy-related NBTE, median (IQR)	1.5 (0.5-4.0)
Autoimmune-related NBTE,	3 (1.25-6.0)
median (IQR)	

AC, anticoagulant; DOAC, direct oral anticoagulants; LMWH, low-molecular weight heparin; NBTE, non-bacterial thrombotic endocarditis; UFH, unfractionated heparin; VKA: vitamin K antagonists.

### Stroke management and treatment outcomes

At the time of stroke presentation, 22% (*n* = 53) of patients were already receiving anticoagulation therapy (Table 2), primarily for prior PE or DVT; 72% (*n* = 38) of these patients were receiving DOACs. After stroke diagnosis, anticoagulation was initiated and/or continued in 70% (*n* = 165) of patients: 53% (*n* = 87) received UFH/LMWH, 16% (*n* = 27) were started on VKA, 20% (*n* = 33) received UFH/LMWH followed by VKA, and 11% (n=18) were treated with DOACs. Additionally, 21% (n=50) of patients underwent valvular surgery.

During a median follow-up of 2.0 months (IQR, 0.5–5.0) after the initial stroke diagnosis, 43% (n=101) of patients experienced recurrent ischemic infarcts and/or stroke syndromes, with 59% (n=59) of these events occurring despite ongoing anticoagulation therapy (Table 2). Recurrent ischemic stroke occurred in 54% (n=83/155) of patients with malignancy (median follow-up 1.5 months [0.5-4.0]) and 32% (n=17/53) of those with autoimmune disease (median follow-up 3.0 months [1.25-6.0]). Recurrent ischemic stroke or infarcts were observed in 56% (*n* = 10/18) of patients receiving DOACs, 29% (*n* = 35/120) of those receiving UFH/LMWH, and 18% (*n* = 11/60) of those receiving VKA. Major bleeding complications occurred in 14% (*n* = 34) of patients following anticoagulation, including intracerebral hemorrhage in 6% (*n* = 21). Overall, 49% (*n* = 115) of patients died after the initial stroke and NBTE diagnosis, with a median time to death of 1.0 month (IQR, 0.35–2.0). Of these deaths, 83% (*n* = 95/115) occurred during hospitalization. Mortality was 17% (*n* = 10/58) among patients with autoimmune-related NBTE compared with 61% (n=100/163) among those with malignancy-related NBTE.

### Predictors of recurrent stroke and all-cause mortality

*All-cause mortality:* In univariate and multivariable regression analyses (Table 3), malignancy-associated NBTE (adjusted odds ratio [aOR], 10.3; 95% CI, 4.0–26.3; *P* < 0.001) and presence of metastases (aOR, 3.9; 95% CI, 2.0–7.7; *P* < 0.001) were independently associated with increased mortality. Conversely, autoimmune-associated NBTE (aOR, 0.1; 95% CI, 0.05–0.33; *P* < 0.001), VKA therapy after stroke diagnosis (aOR, 0.08; 95% CI, 0.03–0.21; *P* < 0.001), and valvular treatment (aOR, 0.4; 95% CI, 0.2–0.8; *P* = 0.013) were associated with lower mortality.

**Table 3:** Univariate and multivariate regression to identify predictors of all-cause mortality and recurrent ischemic stroke.

Variable	Univariate regression (odds ratio 95% CI, *p*-value)	Multivariate regression (odds ratio 95% CI, *p*-value)
**Malignant etiology**		
All-cause mortality	**5.8 (3.0-11.1)**, ***P*** **<** **0.001**	**10.3 (4.0-26.3)**, ***P*** **<** 0.001^*^
Recurrent ischemic stroke	**3.0 (1.6-5.6)**, ***P*** **<** **0.001**	**2.8 (1.4-5.7)**, ***P*** **<** 0.001^*^
Recurrent ischemic stroke despite anticoagulation	**6.7 (2.5-18.2)**, ***P*** **<** **0.001**	**6.2 (2.1-18.3)**, ***P*** **<** 0.001^*^
**Presence of metastases**		
All-cause mortality	**3.9 (2.2-6.8)**, ***P*** **<** **0.001**	**3.9 (2.0-7.7)**, ***P*** **<** 0.001^*^
Recurrent ischemic stroke	**2.5 (1.4-4.3)**, ***P*** **=** **0.001**	**2.3 (1.3-4.2)**, ***P*** **=** 0.006^*^
Recurrent ischemic stroke despite anticoagulation	**2.7 (1.4-5.3)**, ***P*** **=** **0.003**	**2.4 (1.2-4.9)**, ***P*** **=** 0.014^*^
**Autoimmune etiology**		
All-cause mortality	**0.1 (0.07-0.3)**, ***P*** **<** **0.001**	**0.1 (0.05-0.33)**, ***P*** **<** **0.001** ‡
Recurrent ischemic stroke	**0.5 (0.2-0.9)**, ***P*** **=** **0.022**	0.56 (0.3-1.1), *P* = 0.107 ‡
Recurrent ischemic stroke despite anticoagulation	**0.2 (0.09-0.6)**, ***P*** **=** **0.001**	**0.26 (0.1-0.7)**, ***P*** **=** **0.009** ‡
**Anticoagulation after Stroke**		
All-cause mortality	**0.1 (0.04-0.3)**, ***P*** **<** **0.001**	**0.09 (0.04-0.24)**, ***P*** **<** **0.001**^**¶**^
Recurrent ischemic stroke despite anticoagulation	0.9 (0.2-3.3), *P* = 0.905	NA
**Valvular treatment**		
All-cause mortality	**0.4 (0.2-0.7)**, ***P*** **=** **0.003**	**0.4 (0.2-0.8)**, ***P*** **=** **0.013**^**§**^
Recurrent ischemic stroke	0.8 (0.4-1.5), *P* = 0.396	NA
Recurrent ischemic stroke despite anticoagulation	0.8 (0.4-1.7), *P* = 0.577	NA
**UFH/LMWH**		
All-cause mortality	0.7 (0.4-1.2), *P* = 0.171	NA
Recurrent ischemic stroke despite anticoagulation	0.8 (0.4-1.7), *P* = 0.576	NA
**VKA**		
All-cause mortality	**0.05 (0.02-0.13)**, ***P*** **<** **0.001**	**0.08 (0.03-0.21)**, ***P*** **<** **0.001**^**¶**^
Recurrent ischemic stroke despite anticoagulation	**0.3 (0.1-0.8)**, ***P*** **=** **0.013**	0.8 (0.3-3.0), *P* = 0.818
**DOACs**		
All-cause mortality	0.7 (0.3-1.9), *P* = 0.498	NA
Recurrent ischemic stroke despite anticoagulation	**4.0 (1.3-11)**, ***P*** **=** **0.012**	**5.2 (1.4-18.8)**, ***P*** **=** **0.012**^**¶**^

^*^Adjusted for age, gender, anticoagulation treatment after stroke diagnosis, and valvular treatment

^¶^ Adjusted for age, gender, malignant etiology, and autoimmune etiology

^§^ Adjusted for age, gender, malignant etiology, autoimmune etiology

^‡^Adjusted for age, gender, anticoagulation following NBTE/stroke diagnosis, and valvular treatment.

Abbreviations: DOAC: direct oral anticoagulants; LMWH: low-molecular weight heparin; NA: not applicable; UFH: unfractionated heparin; VKA: vitamin K antagonists. ^*^Statistically significant with *P* < 0.05.

*Recurrent stroke:* Malignancy-related NBTE and presence of metastases were independently associated with increased risk of recurrent stroke (aOR, 2.8; 95% CI, 1.4–5.7; *P* < 0.001 and aOR, 2.3; 95% CI, 1.3–4.2; *P* = 0.006, respectively) and recurrent stroke despite anticoagulation (aOR, 6.2; 95 % CI, 2.1–18.3; *P* < 0.001 and aOR, 2.4; 95% CI, 1.2–4.9; *P* = 0.014, respectively). DOAC use was also associated with increased risk of recurrent stroke (aOR, 5.2; 95% CI, 1.4–18.8; *P*=0.012).

## Discussion

This systematic review sought to describe the clinical characteristics, neuroimaging findings, management strategies, and outcomes of stroke patients with NBTE published in prior case reports and series, and is to our knowledge, the most comprehensive analysis to date. Our findings not only reaffirm current understanding but also offer new insights into the therapeutic approaches and prognostic outcomes of NBTE-related stroke while acknowledging the limitations of such review.

**Figure 3:**
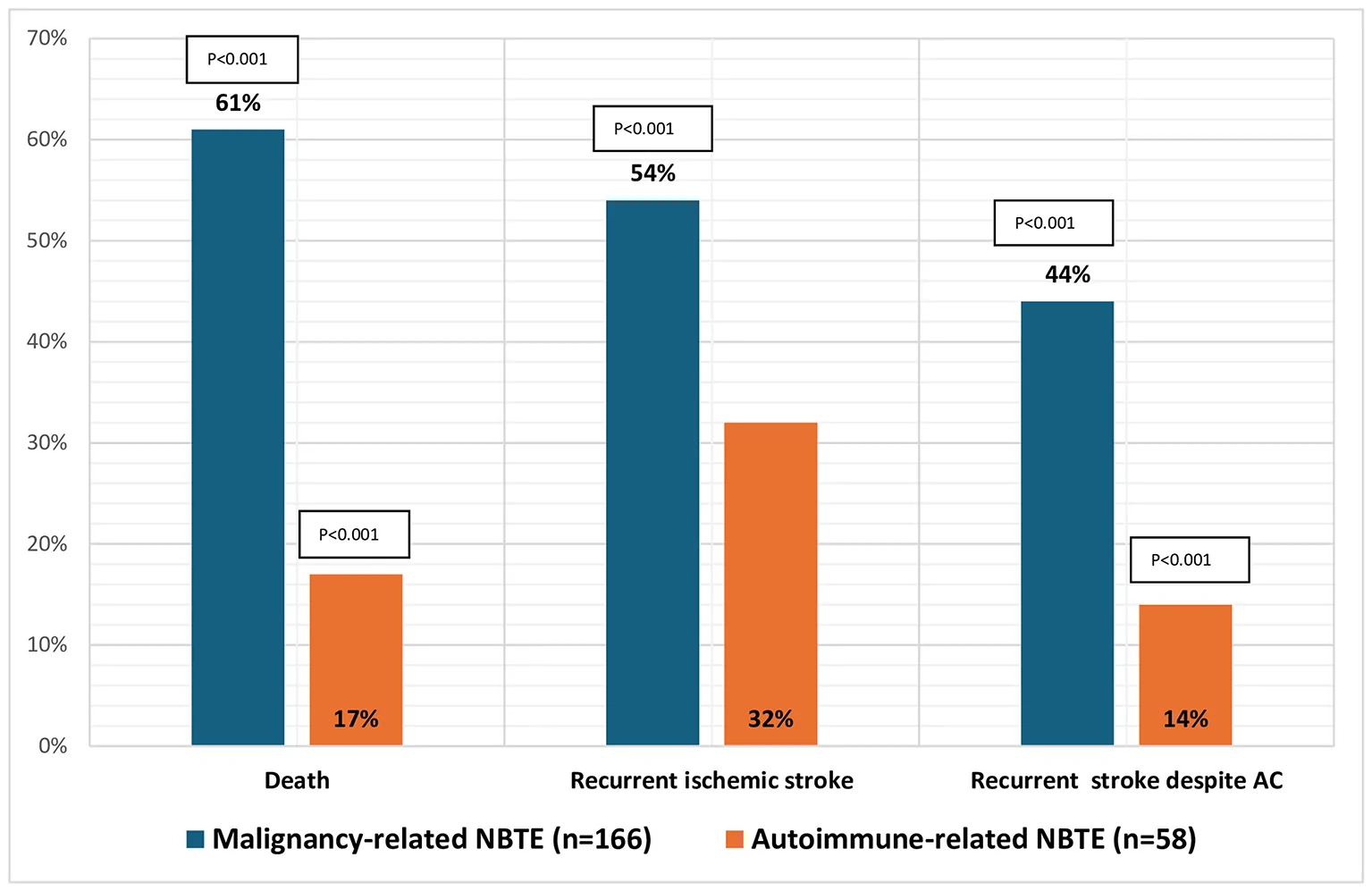
Risk of death, recurrent stroke, and recurrent stroke despite anticoagulation among patients with malignancy-related NBTE compared with autoimmune-related NBTE. AC, anticoagulation; NBTE, non-bacterial thrombotic endocarditis.

Though our study showed that neuroimaging revealed ischemic infarcts affecting multiple vascular territories (>2) in 66% of patients, 18% and 14% of patients had infarcts limited to single and two vascular territories, respectively. Notably, similar findings were reported in a recent study of 84 patients with cancer-related NBTE complicated by stroke, where 16% had infarcts restricted to a single territory (Oakley et al., 2025). In agreement with previous studies, (Patrzalek et al., 2024; Rahouma et al., 2023) malignancy, particularly lung and gastrointestinal (including pancreatic), was the most frequently identified underlying etiology of NBTE. Patients with malignancy demonstrated significantly higher rates of recurrent stroke (54%) and all-cause mortality (61%) (Figure 3). These findings are consistent with reports by Alhuarrat et al. (2024) and Zmaili et al. (2021), which also linked malignancy-related NBTE to increased risks of embolic complications and mortality, respectively. In contrast, a recent single-center study by Patrzałek et al. involving 115 patients with cancer-related NBTE reported a markedly lower 2 year stroke recurrence rate of 12.4%.^1^° The higher recurrence rates observed in our analysis likely reflect publication bias, as case reports and case series with poorer outcomes are more commonly reported. Nonetheless, our findings highlight the importance of maintaining a high index of suspicion for NBTE in patients with active or recent malignancy who present with stroke. Conversely, autoimmune-related NBTE had significantly lower mortality risk (17%) and did not demonstrate a significant increase in stroke recurrence, suggesting a more favorable prognosis in this subgroup compared to those associated with malignant etiology (Figure 3). These contrasting outcomes likely reflect differences in the underlying pathophysiology and may suggest greater responsiveness to anticoagulation in autoimmune-related NBTE.

Anticoagulation remains a cornerstone in the management of NBTE, and this review provides hypothesis-generating observations regarding therapeutic considerations. Although recurrent strokes were observed in 43% of patients in this cohort, 59% of whom were already receiving anticoagulation, VKA anticoagulants were associated with lower mortality. Treatment with DOACs was associated with increased risk of recurrent ischemic infarcts. However, these findings should be interpreted cautiously because anticoagulant selection was not randomized and may have been influenced by malignancy status, thrombotic burden, bleeding risk, prognosis, indication for anticoagulation, and survivorship. Although a recent large retrospective cohort study involving approximately 3,100 NBTE patients found no statistically significant differences in thromboembolic outcomes across different anticoagulant classes (UFH/LMWH, VKA, or DOACs), (Tran et al., 2025) our findings raise concern about DOAC effectiveness in selected patients with NBTE-related stroke and support the need for prospective comparative studies rather than definitive treatment conclusions.

Approximately 50% of patients in this review died within a median of 30 days [IQR:10–60] following the diagnosis of NBTE and stroke—a finding consistent with a recent review of 144 NBTE patients, in which 67% died within a median of 15 days [IQR: 2.7–56] (Rahouma et al., 2023). Most of these deaths (83%) in our review occurred during the initial hospitalization. These findings highlight a narrow therapeutic window associated with NBTE and emphasize the critical need for early recognition and timely intervention.

This review has inherent limitations, primarily related to the nature of its source material. First, reliance on case reports and case series introduces publication and selection biases and limits generalizability, particularly as 70% of included cases were classified as either intermediate or high risk of bias. Second, the protocol was not prospectively registered, although eligibility criteria, outcomes, and data extraction fields were predefined and PRISMA recommendations were followed. Third, inconsistent reporting of follow-up duration and timing of death across heterogeneous case reports resulted in a short median follow-up of 2.0 months (IQR 0.5–5.0), which likely biases the findings toward acute inpatient outcomes while potentially underrepresenting longer-term events, particularly among autoimmune NBTE subgroups. Moreover, our multivariable regression models did not account for important variables like cancer staging and performance status which might have led to differential outcomes between malignancy-related vs. autoimmune-related NBTE cases, and though we limited each model not to adjust for >4 variables simultaneously, model overfitting can remain as another potential limitation. Fourth, statistically significant associations involving anticoagulant class, including VKA, should be interpreted cautiously because treatment allocation was non-randomized and likely affected by confounding by indication, cancer severity, bleeding risk, clinician preference, survival bias, and misclassification bias. The association between DOAC use and recurrent stroke is especially limited by the small number of patients who received DOACs after NBTE and stroke diagnosis (*n* = 18) and possible misclassification bias. Finally, the relatively small sample size likely contributed to wide confidence intervals in multivariable analyses. Larger prospective, multicenter studies are needed to validate these findings and optimize management strategies for NBTE-related stroke.

## Conclusion

NBTE should be considered in stroke patients with minimal vascular risk factors, particularly those with malignancy or autoimmune disease and multifocal infarcts involving multiple vascular territories. Malignancy-associated NBTE presenting with stroke was associated with poor outcomes, including high mortality and recurrent stroke, whereas autoimmune-related NBTE appeared to have a more favorable prognosis. DOAC therapy was associated with increased stroke recurrence in this patient-level synthesis, but this finding should be regarded as hypothesis-generating because of potential confounding, small sample size, and the case-report evidence base. Larger prospective studies are needed to validate these findings and define optimal anticoagulation strategies.

## Data Availability

The original contributions presented in the study are included in the article/Supplementary material, further inquiries can be directed to the corresponding author.
