# Supplementary material for: Stroke in non-bacterial thrombotic endocarditis: a patient-level systematic review of clinical characteristics, management, and outcomes
Source: Front Stroke. 2026 Aug 14;5:1915602. doi: 10.3389/fstro.2026.1915602 (PMC13521837; doi:10.3389/fstro.2026.1915602)
Supplement: Supplementary file 1 [file Data_Sheet_1.docx]

**Search Methodology**

1. **Ovid MEDLINE**
   - **Database: Ovid MEDLINE(R) 1946 to Present and Epub Ahead of Print, In-Process & Other Non-Indexed Citations and Ovid MEDLINE(R) Daily**
     **Search Strategy:**
     **1**  Endocarditis, Non-Infective/ or nonbacterial thrombotic endocarditis.mp. (477)
     **2**  marantic endocarditis.mp. or Endocarditis, Non-Infective/ (255)
     **3**  1 or 2 (530)
     **4**  stroke.mp. or Stroke/ (376388)
     **5**  Ischemic Stroke/ or Cerebral Infarction/ (33997)
     **6**  hemorrhagic stroke.mp. or Hemorrhagic Stroke/ or Cerebral Hemorrhage/ (42651)
     **7**  brain ischemia.mp. or Brain Ischemia/ (65892)
     **8**  brain bleed.mp. (29)
     **9**  4 or 5 or 6 or 7 or 8 (442950)
     **10**  3 and 9 (171)
2. Embase
   - **Database: Embase <1974 to 2025 August 01>**
     **Search Strategy:**
     **1**  non infective endocarditis/ or nonbacterial thrombotic endocarditis.mp. (616)
     **2**  Marantic endocarditis.mp. or non infective endocarditis/ (316)
     **3**  1 or 2 (720)
     **4**  stroke.mp. (564755)
     **5**  ischemic stroke.mp. or ischemic stroke/ (118588)
     **6**  hemorrhagic stroke.mp. (11533)
     **7**  brain ischemia.mp. or brain ischemia/ (161001)
     **8**  brain hemorrhage/ or brain bleed.mp. (132704)
     **9**  4 or 5 or 6 or 7 or 8 (701826)
     **10**  3 and 9 (364)
3. PubMed, Web of Science and PsychINFO
   - Similar search criteria were utilized and retrieved articles were the following
     - PubMed (n=212)
     - Web of Science (n=194)
     - PsychINFO (n=8)

**Articles included in this Systematic Review**

1. Gromke M, Beard B. Non-bacterial Thrombotic Endocarditis Related to Squamous Cell Carcinoma of the Cervix. *Cureus*. 2023;15(7):e42128. Published 2023 Jul 19. doi:10.7759/cureus.42128
2. Akiki E, Ahmad A, El-Am EA, Casanegra AI, Klarich KW, Kurmann R. Case report: New is not always better: treatment of non-bacterial thrombotic endocarditis. *Front Cardiovasc Med*. 2023;10:1208190. Published 2023 Jul 12. doi:10.3389/fcvm.2023.1208190
3. Seo JS. Cerebral infarction related to nonbacterial thrombotic endocarditis in a middle-aged woman with uterine adenomyosis: A case report. *Medicine (Baltimore)*. 2023;102(22):e33871. doi:10.1097/MD.0000000000033871
4. Kovacs J, Khashan A, Kasanga S, Yousaf S, Feingold A. Nonbacterial Thrombotic Endocarditis of the Mitral Valve With Echocardiographic Appearances Mimicking a Papillary Fibroelastoma in a Middle-Aged Female Patient. *Cureus*. 2023;15(4):e37540. Published 2023 Apr 13. doi:10.7759/cureus.37540
5. Zaher W, Balland A, De Cubber M, Sorgente A. Thrombosis of Chiari's network in the setting of non-bacterial thrombotic endocarditis occurring under non-vitamin K antagonist oral anticoagulation: a case report. *Eur Heart J Case Rep*. 2023;7(5):ytad227. Published 2023 May 3. doi:10.1093/ehjcr/ytad227
6. Svantner J, Lavanchy L, Labouchère A. Recurrent embolic strokes due to antiphospholipid syndrome and non-bacterial thrombotic endocarditis in a patient with basal cell carcinoma. *J Cardiothorac Surg*. 2023;18(1):177. Published 2023 May 11. doi:10.1186/s13019-023-02266-6
7. Karan A, Guo HJ, Omar M, Streit S. Extensive Nonbacterial Thrombotic Endocarditis with Recurrent Strokes as a Manifestation of Diffuse Scleroderma. *Am J Med*. 2023;136(7):e142-e143. doi:10.1016/j.amjmed.2023.02.016
8. Karameh M, Golomb M, Arad A, Kalmnovich G, Herzog E. Multi-Valvular Non-bacterial Thrombotic Endocarditis Causing Sequential Pulmonary Embolism, Myocardial Infarction, and Stroke: A Case Report and Literature Review. *Cureus*. 2022;14(12):e32261. Published 2022 Dec 6. doi:10.7759/cureus.32261
9. Benedetti M, Morroni S, Fiaschini P, Coiro S, Savino K. Nonbacterial Thrombotic Endocarditis with Multiple Systemic Emboli in a Patient with Primary Lung Cancer. *J Cardiovasc Echogr*. 2022;32(2):129-131. doi:10.4103/jcecho.jcecho_68_21
10. Babici D, Johansen PM, Rodriguez-Hernandez A, Sommerville S, Snelling B, Miller TD. Recurrent Cardioembolic Strokes Due to Marantic Endocarditis As the Initial Presentation of Colorectal Adenocarcinoma. *Cureus*. 2022;14(4):e24183. Published 2022 Apr 16. doi:10.7759/cureus.24183
11. McCullough J, McCullough J, Kaell A. A Patient's Six-Month Journey From Low Sodium to Blue Toes to Stroke: Non-infective Thrombotic Endocarditis Due to Non-small Cell Lung Cancer. *Cureus*. 2022;14(3):e23235. Published 2022 Mar 16. doi:10.7759/cureus.23235
12. Van Herck J, Thoen H, Delens C, Voet J. Multi-territory stroke preceded by pulmonary embolism with asymptomatic coronavirus disease 2019: a case report. *Eur Heart J Case Rep*. 2021;5(12):ytab471. Published 2021 Nov 19. doi:10.1093/ehjcr/ytab471
13. Harris AZ, Ternouth I, Lallu BD. Case report: marantic endocarditis in renal cell carcinoma: nephrectomy a treatment. *Eur Heart J Case Rep*. 2021;5(11):ytab437. Published 2021 Nov 4. doi:10.1093/ehjcr/ytab437
14. Spurgeon L, Ispoglou S. Non-bacterial thrombotic endocarditis in pancreatic cancer and other high-risk malignancies: the case for prophylactic treatment. *Oxf Med Case Reports*. 2021;2021(11):omab110. Published 2021 Nov 25. doi:10.1093/omcr/omab110
15. Yao JV, Joshi SB, Morgan JG, Lee MG. An unusual case of non-infective endocarditis in undiagnosed antiphospholipid syndrome. *Med J Aust*. 2021;215(7):311-312.e1. doi:10.5694/mja2.51246
16. Savarapu P, Abdelazeem B, Isa S, Baral N, Hassan M. Cancer-Related Non-Bacterial Thrombotic Endocarditis Presenting as Acute Ischemic Stroke. *Cureus*. 2021;13(5):e14953. Published 2021 May 11. doi:10.7759/cureus.14953
17. Panicucci E, Bruno C, Ferrari V, Suissa L. Recurrence of ischemic stroke on direct oral anticoagulant therapy in a patient with marantic endocarditis related to lung cancer. J Cardiol Cases. 2020;23(5):242-245. Published 2020 Nov 24. doi:10.1016/j.jccase.2020.11.010
18. Binet Q, Goffinet C, Etogo-Asse FE, Shaza L. Nonbacterial thrombotic endocarditis in a patient with gastric cancer and SARS-CoV-2 infection. *Clin J Gastroenterol*. 2021;14(4):1031-1035. doi:10.1007/s12328-021-01412-8
19. Miller ES, Hussein HM, Gertner E. A 56-Year-Old Woman with Recurrent Strokes: A Clear Case with a Therapeutic Dilemma. *Case Rep Neurol*. 2021;13(1):100-107. Published 2021 Feb 15. doi:10.1159/000512126
20. Ilyas S, Lang W, Belani N, Stockwell P. Non-Bacterial Thrombotic Endocarditis as a Cause of Cryptogenic Stroke in Malignancy. *R I Med J (2013)*. 2021;104(2):63-66. Published 2021 Mar 1.
21. Holt JN. A case of multifocal stroke-the first presentation of underlying ovarian malignancy. *J Surg Case Rep*. 2021;2021(1):rjaa550. Published 2021 Jan 31. doi:10.1093/jscr/rjaa550
22. Kijpaisalratana N, Chutinet A, Travanichakul S, et al. Nonbacterial Thrombotic Endocarditis Related to Adenocarcinoma of the Uterine Cervix. *Case Rep Neurol*. 2020;12(Suppl 1):183-188. Published 2020 Dec 14. doi:10.1159/000507277
23. Tamura Y, Sakata K, Terada K, Usui S, Kawashiri MA, Takamura M. Treatment with a Direct Oral Anticoagulant for Nonbacterial Thrombotic Endocarditis. *Intern Med*. 2021;60(12):1881-1885. doi:10.2169/internalmedicine.6368-20
24. Patel MJ, Elzweig J. Non-bacterial thrombotic endocarditis: a rare presentation and literature review. *BMJ Case Rep*. 2020;13(12):e238585. Published 2020 Dec 13. doi:10.1136/bcr-2020-238585
25. Basnet S, Stauffer T, Jayswal A, Tharu B. Recurrent nonbacterial thrombotic endocarditis and stroke on anticoagulation. *J Community Hosp Intern Med Perspect*. 2020;10(5):466-469. Published 2020 Sep 3. doi:10.1080/20009666.2020.1791028
26. Wang J, Monga N, Mopala P, Husnain M. Development of Nonbacterial Thrombotic Endocarditis While on Systemic Anticoagulation in Pancreatic Cancer: A Case Report. *Cureus*. 2020;12(10):e10967. Published 2020 Oct 15. doi:10.7759/cureus.10967
27. Galarza Fortuna GM, Singh A, Jacobs A, Ugalde I. Lung Adenocarcinoma Presenting as Multiple Thromboembolic Events: A Case Report and Review of the Literature. *J Investig Med High Impact Case Rep*. 2020;8:2324709620969482. doi:10.1177/2324709620969482
28. Collienne M, Kuhns M, Hauber HP, Arnold D. Severe Nonbacterial Thrombotic Endocarditis Resulting in Aortic Valve Insufficiency and Multiple Small Strokes in a Patient With Metastatic Pancreatic Cancer: Lessons for Optimal Management. *Clin Colorectal Cancer*. 2020;19(4):e258-e261. doi:10.1016/j.clcc.2020.06.001
29. Al-Ghamdi B, Alkaf FA, Al-Anazi MM, et al. Nonbacterial thrombotic endocarditis as an initial presentation of advanced cholangiocarcinoma in a young patient: A case report. *SAGE Open Med Case Rep*. 2020;8:2050313X20945896. Published 2020 Aug 11. doi:10.1177/2050313X20945896
30. Sánchez Quirós B, Ruiz López N, López Herrero R, Bartolomé Bartolomé C. Marantic endocarditis. Endocarditis marántica. *Rev Esp Anestesiol Reanim (Engl Ed)*. 2020;67(4):208-211. doi:10.1016/j.redar.2020.01.005
31. Kaufmann CC, Wessely E, Huber K. Non-bacterial thrombotic endocarditis in the context of pulmonary adenocarcinoma: a case report. *Eur Heart J Case Rep*. 2020;4(1):1-5. Published 2020 Feb 10. doi:10.1093/ehjcr/ytaa008
32. Nadkarni N, Lee YJ, Hoefen R, Alweis R. Cholangiocarcinoma Manifesting as Non-Bacterial Thrombotic Endocarditis in a Young Patient. *Am J Med*. 2020;133(8):e396-e398. doi:10.1016/j.amjmed.2019.11.033
33. Shoji MK, Kim JH, Bakshi S, Govea N, Marukian N, Wang SJ. Nonbacterial Thrombotic Endocarditis Due to Primary Gallbladder Malignancy with Recurrent Stroke Despite Anticoagulation: Case Report and Literature Review. *J Gen Intern Med*. 2019;34(9):1934-1940. doi:10.1007/s11606-019-05166-5
34. Yasuda K, Ayaki T, Kawabata Y, Murase N, Ohtani R, Nakamura M. *Rinsho Shinkeigaku*. 2019;59(4):195-199. doi:10.5692/clinicalneurol.cn-001252
35. Frola A, Baldessari EM, Museli Sylvester T, Casabé JH. Ceguera cortical como manifestación inicial de endocarditis trombótica no bacteriana [Cortical blindness as initial manifestation of nonbacterial thrombotic endocarditis]. *Medicina (B Aires)*. 2019;79(1):61-63.
36. Moţăţăianu A, Maier S, Gothard A, Bajkó Z, Bălaşa R. Severe fatal systemic embolism due to non‑bacterial thrombotic endocarditis as the initial manifestation of gastric adenocarcinoma: case report. *J Crit Care Med (Târgu Mureş)*. 2018;4(2):68‑73. doi:10.2478/jccm‑2018‑0008
37. Yuriditsky E, Torres J, Izmirly PM, Belmont HM. Resolution of large aortic valve vegetations in antiphospholipid syndrome treated with therapeutic anticoagulation: a report of two cases and literature review. *Lupus*. 2018;27(14):2269-2273. doi:10.1177/0961203318804876
38. Fujimoto D, Mochizuki Y, Nakagiri K, Shite J. Unusual rapid progression of non-bacterial thrombotic endocarditis in a patient with bladder cancer despite undergoing intensification treatment with rivaroxaban for acute venous thromboembolism. *Eur Heart J*. 2018;39(43):3907. doi:10.1093/eurheartj/ehy569
39. Soga Y, Taira K, Sugimoto A, et al. Mitral valve nonbacterial thrombotic endocarditis: a rare multi-surgery-tolerant survivor of Trousseau's syndrome. *Surg Case Rep*. 2018;4(1):104. Published 2018 Aug 29. doi:10.1186/s40792-018-0513-5
40. Starobinska E, Robinson EA, Brucks E, Scott S. Marantic endocarditis: incidental infarcts leading to diagnosis of pancreatic cancer. *BMJ Case Rep*. 2018;2018:bcr2018224529. Published 2018 Jun 12. doi:10.1136/bcr-2018-224529
41. Micallef S, Mallia Azzopardi C. Antiphospholipid syndrome masquerading as a case of infective endocarditis. *BMJ Case Rep*. 2018;2018:bcr2018224404. Published 2018 Jun 4. doi:10.1136/bcr-2018-224404
42. Salinas G A, Bustamante H L, Lanas Z F, Soto V A, García B MA, Bartolotti H C. Endocarditis marántica como presentación de cáncer de páncreas. Caso clínico [Non-bacterial thrombotic endocarditis. Report of one case]. *Rev Med Chil*. 2017;145(10):1353-1358. doi:10.4067/S0034-98872017001001353
43. Uchino K, Shimizu T, Mizukami H, et al. Nonbacterial Thrombotic Endocarditis Complicated by Cerebral Infarction in a Patient with Adenomyosis with High Serum CA125 Level; A Case Report. *J Stroke Cerebrovasc Dis*. 2018;27(3):e42-e45. doi:10.1016/j.jstrokecerebrovasdis.2017.09.064
44. Kim K, Kim J, Ahn SH, et al. Histopathological Findings of Intracranial Thrombi in Nonbacterial Thrombotic Endocarditis. *J Stroke*. 2017;19(3):367-369. doi:10.5853/jos.2017.00696
45. Sia CH, Lim JS, Poh KK, Chin TM. A classical case of non-bacterial thrombotic endocarditis from pancreatic adenocarcinoma presenting as multiple strokes, myocardial infarction and acute limb ischaemia. *Oxf Med Case Reports*. 2016;2016(11):omw084. Published 2016 Nov 4. doi:10.1093/omcr/omw084
46. Saito M, Asano N, Ota K, et al. *Kyobu Geka*. 2016;69(13):1067-1071.
47. Morimoto S, Tanaka J, Saito Y, et al. Non-bacterial thrombotic endocarditis in a Trousseau syndrome patient with stomach cancer: A case report. *Geriatr Gerontol Int*. 2016;16(10):1171-1172. doi:10.1111/ggi.12694
48. Bhardwaj B, Bajwa A, Sharma A, Towheed A, Sanghani BV, McGhie AI. Nonbacterial thrombotic endocarditis involving both the tricuspid and aortic valves. *Echocardiography*. 2016;33(12):1916-1918. doi:10.1111/echo.13390
49. Detremerie C, Timmermans F, De Pauw M, et al. Stroke due to non-bacterial thrombotic endocarditis as initial presentation of breast invasive ductal carcinoma. *Acta Clin Belg*. 2017;72(4):268-273. doi:10.1080/17843286.2016.1219012
50. Tai ML, Tan EC, Ang CC, Liam CK. Recurrent cerebral infarcts secondary to marantic endocarditis in a patient with adenocarcinoma of the lung. *Singapore Med J*. 2016;57(9):524-525. doi:10.11622/smedj.2016157
51. Guerreiro S, Gouveia C, de Féria RM, Lourenço A, Grenho F. Behind Recurrent Thromboembolic Events. *Eur J Case Rep Intern Med*. 2016;3(6):000413. Published 2016 Aug 3. doi:10.12890/2016_000413
52. Gundersen H, Moynihan B. An Uncommon Cause of Stroke: Non-bacterial Thrombotic Endocarditis. *J Stroke Cerebrovasc Dis*. 2016;25(10):e163-e164. doi:10.1016/j.jstrokecerebrovasdis.2015.11.009
53. Liang LW, Perez AR, Cangemi NA, Young RJ, Makker V. Nonbacterial thrombotic endocarditis with embolic cerebral vascular accidents in a patient with advanced, recurrent clear cell carcinoma of the ovary: A case report. *Gynecol Oncol Rep*. 2016;16:25-27. Published 2016 Mar 17. doi:10.1016/j.gore.2016.03.003
54. Choi JH, Park JE, Kim JY, Kang T. Non-Bacterial Thrombotic Endocarditis in a Patient with Rheumatoid Arthritis. *Korean Circ J*. 2016;46(3):425-428. doi:10.4070/kcj.2016.46.3.425
55. Mitma AA, Varghese JG, Witt D, Zarich SW. Stroke and a valvular lesion in a patient with stage IV non-small cell lung cancer. *BMJ Case Rep*. 2016;2016:bcr2016215317. Published 2016 May 31. doi:10.1136/bcr-2016-215317
56. Yasutake H, Sugano Y, Ikeda Y, et al. First Case Report of the Antemortem Diagnosis of Nonbacterial Thrombotic Endocarditis of a Mechanical Prosthetic Valve. *Intern Med*. 2016;55(3):255-257. doi:10.2169/internalmedicine.55.5470
57. De Langhe E, Seghers A, Demaerel P, Verschueren P, Lemmens R. Non-infective endocarditis with systemic embolization and recurrent stroke in systemic sclerosis. *Rheumatology (Oxford)*. 2016;55(3):589-591. doi:10.1093/rheumatology/kev381
58. Lin YK, Lee JT, Yang FC, et al. Systemic embolic events with nonbacterial thrombotic endocarditis as manifestations of recurrent ovarian clear cell carcinoma. *Taiwan J Obstet Gynecol*. 2015;54(5):625-628. doi:10.1016/j.tjog.2015.08.021
59. Tsai CC, Wu MN. Frequent Ischemic Stroke as First Manifestation of Occult Colon Cancer: A Rare Case. *Am J Case Rep*. 2015;16:723-727. Published 2015 Oct 12. doi:10.12659/ajcr.895130
60. Dokuni K, Matsumoto K, Tanaka H, Okita Y, Hirata K. A case of non-infective endocarditis accompanied by multiple cerebral infarctions and severe mitral regurgitation as initial presentation of primary antiphospholipid syndrome. *Eur Heart J Cardiovasc Imaging*. 2015;16(5):572. doi:10.1093/ehjci/jeu325
61. Elboudwarej O, Wei J, Siegel R. Diagnosis of an aortic valvular lesion. Non-bacterial thrombotic endocarditis (NBTE). *Heart*. 2015;101(9):719-726. doi:10.1136/heartjnl-2014-306723
62. Wang LW, Noël B, Descloux E, Baron DW. Antiphospholipid syndrome: an important differential diagnosis for culture-negative endocarditis. *Am J Med*. 2015;128(3):250-253. doi:10.1016/j.amjmed.2014.11.010
63. Yagi T, Takahashi K, Tanikawa M, Seki M, Abe T, Suzuki N. Fatal intracranial hemorrhage after intravenous thrombolytic therapy for acute ischemic stroke associated with cancer-related nonbacterial thrombotic endocarditis. *J Stroke Cerebrovasc Dis*. 2014;23(8):e413-e416. doi:10.1016/j.jstrokecerebrovasdis.2014.04.004
64. Yamane A, Sadahiro H, Goto H, et al. Multiple ischemic strokes caused by nonbacterial thrombotic endocarditis because of gallbladder cancer: a case report. *J Stroke Cerebrovasc Dis*. 2014;23(6):1727-1729. doi:10.1016/j.jstrokecerebrovasdis.2013.12.031
65. Moustafa S, Patton DJ, Balon Y, Kidd WT, Alvarez N. Mitral valve surgery for marantic endocarditis and multiple cerebral embolisation. *Heart Lung Circ*. 2013;22(7):545-547. doi:10.1016/j.hlc.2012.11.007
66. Devulapalli S, Pinto N, Gandothra C, Jayam-Trouth A, Kurukumbi M. A rare case of occipital stroke as a consequence of nonbacterial thrombotic endocarditis in ovarian clear cell carcinoma: a case report. *Case Rep Neurol*. 2012;4(1):84-91. doi:10.1159/000338811
67. Sakima H, Isa K, Kokuba K, et al. Recurrent embolic stroke due to nonbacterial thrombotic endocarditis followed by transesophageal echocardiography. *Arch Neurol*. 2011;68(12):1604-1605. doi:10.1001/archneurol.2011.687
68. Scalia GM, Tandon AK, Robertson JA. Stroke, aortic vegetations and disseminated adenocarcinoma--a case of marantic endocarditis. *Heart Lung Circ*. 2012;21(4):234-236. doi:10.1016/j.hlc.2011.08.003
69. Arvold ND, Hsu L, Chen WY, Benzaquen LR, Weiss SE. Marantic endocarditis with cardioembolic strokes mimicking leptomeningeal metastases in breast cancer. *J Clin Oncol*. 2011;29(29):e743-e746. doi:10.1200/JCO.2011.36.4190
70. Elikowski W, Lazowski S, Małek M, Wróblewski D, Kuźniacka-Kaczor E, Skowroński M. Nietypowy obraz zylnych powikłań zakrzepowo-zatorowych i zatorowość systemowa u chorego z nieinfekcyjnym zakrzepowym zapaleniem wsierdzia w przebiegu raka trzustki [Uncommon presentation of venous thromboembolism and fatal systemic emboli in a patient with nonbacterial thrombotic endocarditis due to pancreatic cancer]. *Kardiol Pol*. 2010;68(6):678-683.
71. Pons F, Poyet R, Daranda E, et al. Accidents cérébraux ischémiques sous héparine révélateurs d'endocardites marastiques [Ischemic strokes as a presenting feature of marantic endocarditis despite heparin treatment]. *Ann Cardiol Angeiol (Paris)*. 2011;60(4):233-235. doi:10.1016/j.ancard.2010.07.013
72. Dupeux S, Bricaire L, Bosquet A, Pouchot J, Capron L. Endocardite marastique et adénocarcinome gastrique [Nonbacterial thrombotic endocarditis and gastric carcinoma]. *Rev Med Interne*. 2008;29(8):673-675. doi:10.1016/j.revmed.2007.12.023
73. Joshi SB, Richards MJ, Holt DQ, Yan BP, Aggarwal A. Marantic endocarditis presenting as recurrent arterial embolisation. *Int J Cardiol*. 2009;132(1):e14-e16. doi:10.1016/j.ijcard.2007.07.106
74. Scozzafava J, Hussain MS, Ahmed SN, Khan K. Recurrent strokes in a 46-year-old woman: rapidly progressive nonbacterial thrombotic endocarditis. *CMAJ*. 2006;175(9):1055. doi:10.1503/cmaj.060499
75. Letsas KP, Filippatos GS, Kounas SP, Pappas LK, Gavrielatos G, Kardaras F. Primary antiphospholipid syndrome and factor V Leiden mutation in a young patient with non-bacterial thrombotic endocarditis and transient ischemic stroke. *Thromb Haemost*. 2005;94(6):1331-1332. doi:10.1160/TH05-06-1331
76. Royter V, Cohen SN. Recurrent embolic strokes and cardiac valvular disease in a patient with non-small cell adenocarcinoma of lung. *J Neurol Sci*. 2006;241(1-2):99-101. doi:10.1016/j.jns.2005.10.008
77. Rabinstein AA, Giovanelli C, Romano JG, Koch S, Forteza AM, Ricci M. Surgical treatment of nonbacterial thrombotic endocarditis presenting with stroke. *J Neurol*. 2005;252(3):352-355. doi:10.1007/s00415-005-0660-z
78. Chen L, Li Y, Gebre W, Lin JH. Myocardial and cerebral infarction due to nonbacterial thrombotic endocarditis as an initial presentation of pancreatic adenocarcinoma. *Arch Pathol Lab Med*. 2004;128(11):1307-1308. doi:10.5858/2004-128-1307-MACIDT
79. Lal G, Brennan TV, Hambleton J, Clark OH. Coagulopathy, marantic endocarditis, and cerebrovascular accidents as paraneoplastic features in medullary thyroid cancer--case report and review of the literature. *Thyroid*. 2003;13(6):601-605. doi:10.1089/105072503322238872
80. Sharma S, Mayberry JC, Deloughery TG, Mullins RJ. Fatal cerebroembolism from nonbacterial thrombotic endocarditis in a trauma patient: case report and review. *Mil Med*. 2000;165(1):83-8
81. Vassallo R, Remstein ED, Parisi JE, Huston J 3rd, Brown RD Jr. Multiple cerebral infarctions from nonbacterial thrombotic endocarditis mimicking cerebral vasculitis. *Mayo Clin Proc*. 1999;74(8):798-802. doi:10.4065/74.8.7985.
82. Borowski A, Ghodsizad A, Cohnen M, Gams E. Recurrent embolism in the course of marantic endocarditis. *Ann Thorac Surg*. 2005;79(6):2145-2147. doi:10.1016/j.athoracsur.2003.12.024
83. Gallerini S, Fanucchi S, Sonnoli C, Gelli A, Sartucci F, Orlandi G. Stroke in the young as the clinical onset of non-Hodgkin's lymphoma with paraneoplastic endocarditis. *Eur J Neurol*. 2004;11(6):421-422. doi:10.1111/j.1468-1331.2004.00823.x
84. Van Venrooy, A, Shisler, D, Katzenstein, P. FOUR STROKE ALERTS IN TWO MONTHS: A CASE OF NONBACTERIAL THROMBOTIC ENDOCARDITIS COMPLICATING RHEUMATOID ARTHRITIS. JACC. 2021 May, 77 (18_Supplement_1) 2807.https://doi.org/10.1016/S0735-1097(21)04162-0
85. LUNG ADENOCARCINOMA PRESENTING AS NONBACTERIAL THROMBOTIC ENDOCARDITIS (NBTE) AND COMPENSATED DISSEMINATED INTRAVASCULAR COAGULATION (DIC) WITH EMBOLIC PHENOMENON AL-AHMAD, MAJD et al. CHEST, Volume 162, Issue 4, A1667 - A1668
86. A DELAYED DIAGNOSIS OF NONBACTERIAL THROMBOTIC ENDOCARDITIS IN THE SETTING OF LUNG ADENOCARCINOMA BERNSKOETTER, NICOLE et al. CHEST, Volume164, Issue 4, A4431
87. A TALE OF TWO VALVES: MARANTIC ENDOCARDITIS OF THE TRICUSPID AND MITRAL VALVES WITH DEVASTATING OUTCOME ABUGHAZALEH, SAEED et al. CHEST, Volume 164, Issue 4, A4489 - A4490
88. Verma, A., Bhatta, N., Mishra, D. R., Acharya, A. B., Shahi, R., LImbu, S. H. ., Katuwal, S., & Lama, U. (2021). Mesothelioma with Nonbacterial Thrombotic Endocarditis: A Case Report. *Journal of Nepal Medical Association*, *59*(243), 1170–1173. <https://doi.org/10.31729/jnma.6841>
89. Abstracts from the 2020 Annual Meeting of the Society of General Internal Medicine. *J GEN INTERN MED* **35** (Suppl 1), 1–779 (2020). <https://doi.org/10.1007/s11606-020-05890-3>
90. 441 An Atypical Case of Marantic Endocarditis in Undiagnosed Antiphospholipid Syndrome Yao, J. et al. Heart, Lung and Circulation, Volume 29, S235
91. ADENOCARCINOMA OF THE LUNG PRESENTING AS AN ACUTE STROKE: THE CLINICAL CHALLENGE OF NONBACTERIAL THROMBOTIC ENDOCARDITIS Guiance, Irene Riestra et al. CHEST, Volume 158, Issue 4, A1548
92. Chamsi-Pasha, M.A., Al Emam, A.R.A., Sayyed, S.H. *et al.* An unusual cause of cardioembolic stroke. *Int J Cardiovasc Imaging* **32**, 261–262 (2016). <https://doi.org/10.1007/s10554-015-0764-1>
93. Kanthasamy V, Natarajan I. Rare and challenging case of stroke as a manifestation of non bacterial thrombotic endocarditis in an underlying ovarian clear cell carcinoma. *JRSM Open*. 2016;7(11):2054270416669304. Published 2016 Nov 1. doi:10.1177/2054270416669304
94. Wong SF, Seow J, Profitis K, Johns J, Barnett S, John T. Marantic endocarditis presenting with multifocal neurological symptoms. *Intern Med J*. 2013;43(2):211-214. doi:10.1111/imj.12018
95. Kanthasamy V, Natarajan I. Rare and challenging case of stroke as a manifestation of non bacterial thrombotic endocarditis in an underlying ovarian clear cell carcinoma. *JRSM Open*. 2016;7(11):2054270416669304. Published 2016 Nov 1. doi:10.1177/2054270416669304
96. Yung-Hsiang Hsu, Nonbacterial thrombotic endocarditis, Tzu Chi Medical Journal, Volume 25, Issue 3, 2013, Pages 186-187, ISSN 1016-3190, <https://doi.org/10.1016/j.tcmj.2012.08.007>.
97. Ito S, Yoshitomi H, Pak M, et al. Trousseau syndrome with nonbacterial thrombotic endocarditis in a patient with uterine cancer. *Intern Med*. 2013;52(12):1353-1358. doi:10.2169/internalmedicine.52.9384
98. Abstracts of the Annual Conference of the Asia Pacific Stroke Organization (APSO). Hong Kong, August 30–September 1, 2013. *Cerebrovasc Dis* 1 August 2013; 36 (Suppl. 1): 1–94. <https://doi.org/10.1159/000353795>
99. M Molisana, S Belleggia, G Gizzi, U Ianni, S D’Agostino, M Di Eusanio, A Dello Russo, V Parato, C35 A RARE CASE OF NON–BACTERIAL THROMBOTIC ENDOCARDITIS IN YOUNG WOMAN AFFECTED BY ADVANCED UTERINE CANCER, *European Heart Journal Supplements*, Volume 25, Issue Supplement_D, May 2023, Pages D15–D16, <https://doi.org/10.1093/eurheartjsupp/suad111.034>
100. Dabhad, R, Sunner, G, Ashfaq, A. et al. A RARE PRESENTATION OF NONBACTERIAL THROMBOTIC ENDOCARDITIS DUE TO ENDOMETRIAL ADENOCARCINOMA. JACC. 2023 Mar, 81 (8_Supplement) 3841. <https://doi.org/10.1016/S0735-1097(23)04285-7>
101. Patel, H, Diem, D, Keyes, P. et al. NONBACTERIAL THROMBOTIC ENDOCARDITIS WITH UNDERLYING OVARIAN CARCINOMA RESOLVING WITH ONCOLOGIC TREATMENT. JACC. 2023 Mar, 81 (8_Supplement) 3020. <https://doi.org/10.1016/S0735-1097(23)03464-2>
102. Solis, J, Guajardo, D, Melara, Q. et al. MARANTIC ENDOCARDITIS AND THROMBOTIC STORM IN A YOUNG WOMAN WITH AN UNDERLYING MALIGNANCY. JACC. 2023 Mar, 81 (8_Supplement) 3012.https://doi.org/10.1016/S0735-1097(23)03456-3
103. Louis W Wang, Justin Phan, Priyanka Schuetz, Abdullah Omari, Alasdair J Watson, Rajesh N Subbiah, Splinters in the fingernails, heart and brain: thromboembolic complications of non-bacterial thrombotic endocarditis despite treatment with a direct-acting oral anticoagulant, *Oxford Medical Case Reports*, Volume 2022, Issue 1, January 2022, omab138, <https://doi.org/10.1093/omcr/omab138>
104. Okuchi K, Fujioka M, Iwanaga H, Koshimae N, Sakaki T. Fulminant cerebral infarctions caused by nonbacterial thrombotic endocarditis due to gallbladder cancer. *Acta Neurochir (Wien)*. 1997;139(10):995-996. doi:10.1007/BF01411313
105. Fulham MJ, Gatenby P, Tuck RR. Focal cerebral ischemia and antiphospholipid antibodies: a case for cardiac embolism. *Acta Neurol Scand*. 1994;90(6):417-423. doi:10.1111/j.1600-0404.1994.tb02751.x
106. Fujishima S, Okada Y, Irie K, et al. Multiple brain infarction and hemorrhage by nonbacterial thrombotic endocarditis in occult lung cancer--a case report. *Angiology*. 1994;45(2):161-166. doi:10.1177/000331979404500213
107. Glass JP. The diagnosis and treatment of stroke in a patient with cancer: nonbacterial thrombotic endocarditis (NBTE): a case report and review. *Clin Neurol Neurosurg*. 1993;95(4):315-318. doi:10.1016/0303-8467(93)90108-s
108. Marchandise S, Humblet Y, Lambert M. Cerebrovascular accident in a cancer patient: Consider a nonbacterial thrombotic endocarditis (marantic endocarditis). Case Rep Oncol Med. 2007; 126 (6): 175-178
109. Higes-Pascual F, Tello-Blasco S, Fernández-Santos A, Hernández-García M. Isquemia cerebral secundaria a una endocarditis trombótica no bacteriana como forma de presentación de un tumor ovárico [Cerebral ischaemia secondary to non-bacterial thrombotic endocarditis as the presenting symptom of an ovarian tumour]. *Rev Neurol*. 2005;41(7):404-408.
110. Herrera de Pablo P, Esteban Esteban E, Giménez Soler JV, Pareja Martínez A, Moscoso del Prado J. Endocarditis trombótica no bacteriana como manifestación inicial de neoplasia pulmonar [Nonbacterial thrombotic endocarditis as initial event of lung cancer]. *An Med Interna*. 2004;21(10):495-497. doi:10.4321/s0212-71992004001000007
111. White SJ. Nonbacterial Thrombotic Endocarditis of Bioprosthetic Aortic Valve Presenting as Cardioembolic Stroke in a Patient without Predisposing Systemic Disease. *Case Rep Cardiol*. 2023;2023:5411153. Published 2023 Oct 12. doi:10.1155/2023/5411153
112. Kim B, Kim SH, Kim T. Cerebral Infarcts by Nonbacterial Thrombotic Endocarditis Associated with Adenomyosis: A Case Report. *J Stroke Cerebrovasc Dis*. 2018;27(3):e50-e53. doi:10.1016/j.jstrokecerebrovasdis.2017.10.005
113. Mukai M, Hamada M, Hiwada K, Kokubu T, Nakajo A, Go S. Cerebral and myocardial infarction induced by nonbacterial thrombotic endocarditis in a patient with ovarian cancer: report of a case. *Jpn J Med*. 1988;27(3):321-324. doi:10.2169/internalmedicine1962.27.321
114. George J, Lamb JT, Harriman DG. Cerebral embolism due to non-bacterial thrombotic endocarditis following pregnancy. *J Neurol Neurosurg Psychiatry*. 1984;47(1):79-80. doi:10.1136/jnnp.47.1.79
115. Young RS, Zalneraitis EL. Marantic endocarditis in children and young adults: clinical and pathological findings. *Stroke*. 1981;12(5):635-639. doi:10.1161/01.str.12.5.635
116. Gabelmann V, Grabs F, Diestelmeier S, et al. Quadrivalvular nonbacterial thrombotic endocarditis in a patient with clear cell cervical cancer. *Clin Case Rep*. 2022; 10:e06434. doi: [10.1002/ccr3.6434](https://doi.org/10.1002/ccr3.6434)
117. [Jyotsana Sinha](https://www.atsjournals.org/author/Sinha%2C+Jyotsana) , [Lauren J. Van Scoy](https://www.atsjournals.org/author/van+Scoy%2C+Lauren+J) , [Robert Promisloff](https://www.atsjournals.org/author/Promisloff%2C+Robert) , Adenocarcinoma Of The Lung Presenting With Nonbacterial Thrombotic Endocarditis With Multi-Organ Embolic Phenomenon And Cardiac Tamponade, American Journal of Respiratory and Critical Care Medicine 2012;185:A4397 <https://doi.org/10.1164/ajrccm-conference.2012.185.1_MeetingAbstracts.A4397>
118. Saraswati, U, Abdulhai, F, Kandula, P. et al. MARANTIC ENDOCARDITIS OF A BICUSPID AORTIC VALVE IN A YOUNG MALE PRESENTING WITH STROKE AND ANTIPHOSPHOLIPID SYNDROME. JACC. 2023 Mar, 81 (8_Supplement) 3743. <https://doi.org/10.1016/S0735-1097(23)04187-6>
119. ltaher H, Saleem M, Coulson D*, et al* Non-bacterial thrombotic endocarditis in a patient with high-grade serous ovarian carcinoma diagnostic challenges and management insights *BMJ Case Reports CP* 2025;**18:**e262906
120. Anees, M., Wilber, E. & Phadke, V.K. Elephant in the Room: an Exercise in Clinical Reasoning. *J GEN INTERN MED* **39**, 3349–3353 (2024). <https://doi.org/10.1007/s11606-024-09027-8>
121. Aortic Valve Vegetation due to Nonbacterial Thrombotic Endocarditis in a Patient with Antiphospholipid Antibody Syndrome Bahar, Abdul Rasheed et al. CASE, Volume 8, Issue 7, 386 - 389
122. Barbosa Mateus D, Jesus T. Embolic Stroke Unveiling Lung Cancer: A Case of Cancer-Associated Non-bacterial Thrombotic Endocarditis. *Cureus*. 2025;17(2):e78339. Published 2025 Feb 1. doi:10.7759/cureus.78339
123. Nonbacterial Thrombotic Endocarditis (NBTE) Treated With Crizotinib in Lung Adenocarcinoma Harboring ROS-1 Rearrangement: Two Case Report and Review of Literature Chamorro-Pérez, Jesús et al. Clinical Lung Cancer, Volume 26, Issue 4, e293 - e299
124. The Silent Aortic valve mass. F Correa The American Journal of the Medical Sciences 367(S1) (2024) S1–S212
125. Costa C, Dalal F, Buhari O, Fan J, Costa S. Rapid Embolic Event Chain: An Unusual Presentation of Marantic Endocarditis. *Cureus*. 2025;17(5):e83667. Published 2025 May 7. doi:10.7759/cureus.83667
126. El Koubayati G, Sandakly N, Jabbour I, Haddad F, Khalil M. Ischemic Stroke Revealing Libman-Sacks Endocarditis as the Initial Manifestation of Lupus Erythematosus With Secondary Antiphospholipid Syndrome: A Case Report. *Cureus*. 2024;16(10):e70870. Published 2024 Oct 5. doi:10.7759/cureus.70870
127. Marantic endocarditis and stroke: Case presentation. Faria A. Clinical Medicine, Volume 24, Supplement, 2024, 100084, ISSN 1470-2118, <https://doi.org/10.1016/j.clinme.2024.100084>.
128. Ferrentino F, Campana S, Torri M, Rostagno C. Neuropsychiatric disorder as early clinical presentation of marantic endocarditis in patient with newly diagnosed poorly differentiated esophago-gastric junction adenocarcinoma: A case report. *Clin Case Rep*. 2024;12(4):e8710. Published 2024 Apr 12. doi:10.1002/ccr3.8710
129. Guevara N, Sanchez J, Chapiolkina V, et al. The Interplay of Malignancy and Endocarditis: A Report of a Rare Case of Marantic Endocarditis in Metastatic Lung Adenocarcinoma. *Cureus*. 2024;16(6):e63456. Published 2024 Jun 29. doi:10.7759/cureus.63456
130. Gregory L. Guzik, Joy W. Li, Joshua B. Wiener, Debora S. Bruno; Incidentally Discovered Endocarditis Leading to the Diagnosis of an Epidermal Growth Factor Receptor Mutant Metastatic Pulmonary Malignancy of Occult Primary Tumor. *Case Rep Oncol* 18 June 2024; 17 (1): 686–694. <https://doi.org/10.1159/000539454>
131. Haider, Mobeen, Khalil, Muhammad Hasib, Khalid, Yousra, et al. Marantic Endocarditis- A Rare Paraneoplastic Presentation of Ovarian Carcinoma. Circulation 2023;148
132. Jonsdottir, S. Stroke and heart vale prolapse-Medical Case Laeknabladid 2025;111(3):119-121. 2025
133. Kovacs J, Khashan A, Kasanga S, Yousaf S, Feingold A. Nonbacterial Thrombotic Endocarditis of the Mitral Valve With Echocardiographic Appearances Mimicking a Papillary Fibroelastoma in a Middle-Aged Female Patient. *Cureus*. 2023;15(4):e37540. Published 2023 Apr 13. doi:10.7759/cureus.37540.
134. Mitrani, L. Non-Bacterial Thrombotic Endocarditis and Recurrent Cerebrovascular Accidents in Immune Checkpoint Inhibitor Myocarditis. Circulation 2023;148
135. Mohamed W, Ahmed A, Mansour S, Zlocha V. Left ventricular free-wall rupture: An unusual presentation of non-bacterial thrombotic endocarditis. *Perfusion*. 2022;38(5):1085-1087. doi:[10.1177/02676591221092313](https://doi.org/10.1177/02676591221092313)
136. Muranaka M, Tobino K. Unveiling the Unexpected: Lung Adenocarcinoma Presenting With Penile Mondor's Disease and Multiple Thrombotic Complications. *Cureus*. 2024;16(10):e72610. Published 2024 Oct 29. doi:10.7759/cureus.72610
137. Papakonstantinou, K., Rorris, FP., Stanitsa, N. *et al.* PET-scan in diagnosis of non-bacterial thrombotic endocarditis: a case report. *Egypt Heart J* **76**, 21 (2024). <https://doi.org/10.1186/s43044-024-00452-y>
138. Multi-Valve Libman-Sacks’s Endocarditis-Related Multiple, Massive and Fatal Systemic Embolization. A Case Report and a Review of Diagnostic Work-up”. 2023. *Monaldi Archives for Chest Disease* 94 (3). <https://doi.org/10.4081/monaldi.2023.2729>.
139. Patil R, Yongue C, James L, Zhou F, Saric M, Vaynblat M. Presentation and management of marantic endocarditis: A case series. *Echocardiography*. 2024; 41: 1–12. [**https://doi.org/10.1111/echo.15891**](https://doi.org/10.1111/echo.15891)
140. Rahman, Omar^1^; Ijaz, Muniba^2^; Neha Aamer, Syeda^2^; Siddiqui, Huda^2^; Azhar, Aima^2^; Shabbir, Usama^2^; Nawaz, Maryam^2^; Basnet, Sagarika^2^; Mahak, FNU^2^; Alam, Fatima^2^; Sharif, Aleena^2^. 682: LUNG CANCER-RELATED NONBACTERIAL THROMBOTIC ENDOCARDITIS PRESENTING AS MASSIVE ACUTE ISCHEMIC STROKE. Critical Care Medicine 53(1):, January 2025. | DOI: 10.1097/01.ccm.0001101392.25270.6d
141. Rankin WR, Querin LB, Free M, Martini WA. Nonbacterial Thrombotic Endocarditis in a 78-Year-Old Female With Recurrent Acute Ischemic Stroke: A Case Report. *Cureus*. 2024;16(10):e70887. Published 2024 Oct 5. doi:10.7759/cureus.70887
142. Recuero-Borau J, Masfarré L, Navarro N, Rocha P, Taus Á, Arriola E. Non-bacterial thrombotic endocarditis in *ROS1*-rearranged lung cancer: a report of two cases. *Transl Lung Cancer Res*. 2024;13(1):179-185. doi:10.21037/tlcr-23-412
143. Reis, Catarina Santos, Dias, Francisco, Granja, Bárbara, Matos, Maria Inês, Ribeiro, Ana, Friões, Fernando, Severe Mitral Regurgitation–Induced Acute Heart Failure due to Nonbacterial Thrombotic Endocarditis in a Patient With Urothelial Carcinoma Recurrence, *Case Reports in Oncological Medicine*, 2025, 9938933, 5 pages, 2025. [**https://doi.org/10.1155/crom/9938933**](https://doi.org/10.1155/crom/9938933)
144. Sadar, A.; Arunraj, C.N.; Sreelal, S. Ischemic Stroke: A Case Series Unraveling Benign and Malignant Causes of High Prothrombotic Tendency. European Journal of Cardiovascular Medicine 2025;15(4):964-974. DOI: [10.61336/ejcm/25-04-158](https://dx.doi.org/10.61336/ejcm/25-04-158)
145. Santiago LE, Kujundzic W, Wong S, Swaminath S, Aneja P. An Unusual Case of Nonbacterial Thrombotic Endocarditis Attributable to Malignancy. *Cureus*. 2023;15(9):e44734. Published 2023 Sep 5. doi:10.7759/cureus.44734
146. Tisch C, Ernst D, Falke M, Speicher P, Ziaka M. Systemic embolization due to non-bacterial thrombotic endocarditis: An autopsy case report and mini review of the literature. *SAGE Open Medical Case Reports*. 2024;12. doi:[10.1177/2050313X241229576](https://doi.org/10.1177/2050313X241229576)
147. Vattikonda KS, Peterson CJ, Ayzenbart VI, Rutherford MS. Nonbacterial Thrombotic Endocarditis With Embolic Phenomena Diagnosed by Transesophageal Echocardiogram. *Cureus*. 2023;15(9):e45686. Published 2023 Sep 21. doi:10.7759/cureus.45686
148. Tian-Yu You, Nan-Yao Lee, Ming-Chi Li, Keng-Fu Hsu, Po-Wei Chen, Chun-Min Wang, Wen-Chien Ko, Nonbacterial thrombotic endocarditis mimics acute infective endocarditis in a woman with endometrial cancer, Journal of Microbiology, Immunology and Infection, Volume 57, Issue 4, 2024, Pages 665-667, ISSN 1684-1182, <https://doi.org/10.1016/j.jmii.2024.02.006>
149. Zaman MA, Sohail MO, Sbeitan I, Aldergash SM. Prolonged Antibiotics Versus Prolonged Anticoagulation: A Case Report of Libman-Sacks Endocarditis. *Cureus*. 2025;17(4):e83203. Published 2025 Apr 29. doi:10.7759/cureus.83203
150. Kaneyuki D, Matsuura K, Ueda H, Kohno H, Kanbe M, Matsumiya G. Surgical management of nonbacterial thrombotic endocarditis in malignancy. *Surg Case Rep*. 2017;3(1):60. doi:10.1186/s40792-017-0335-x
151. Sirinvaravong N, Rodriguez Ziccardi MC, Patnaik S, et al. Nonbacterial thrombotic endocarditis in a patient with primary antiphospholipid syndrome. *Oxf Med Case Reports*. 2018;2018(6):omy024. Published 2018 Jun 25. doi:10.1093/omcr/omy024
152. Markides V, Nihoyannopoulos P. Non-bacterial thrombotic endocarditis. *Eur J Echocardiogr*. 2000;1(4):291-294. doi:10.1053/euje.2000.0027
153. Ye T, Wang J, Liao S. Mitral Valve Repair for Isolated Libman-Sacks Endocarditis in a Patient with Primary Antiphospholipid Syndrome. *Int Heart J*. 2021;62(1):181-185. doi:10.1536/ihj.20-260
154. Hashimoto T, Aoki T, Kawabata Y, Owai Y, Matsuda Y, Tamura S. Nonbacterial Thrombotic Endocarditis Associated with Acute Promyelocytic Leukemia: An Autopsy Case Report. *Medicina (Kaunas)*. 2021;57(11):1264. Published 2021 Nov 18. doi:10.3390/medicina57111264
155. Randhawa G, Aslam A, Suarez MJ, Lin YS, Kuhn-Basti M. Non-Bacterial Thrombotic Endocarditis: A Case of Metastatic Pancreatic Cancer Masquerading as Infective Endocarditis. *Cureus*. 2020;12(7):e9103. Published 2020 Jul 9. doi:10.7759/cureus.9103
156. Uchida W, Mutsuga M, Ito H, Oshima H, Usui A. Nonbacterial Thrombotic Endocarditis Associated With Crohn Disease. *Ann Thorac Surg*. 2018;105(5):e199-e201. doi:10.1016/j.athoracsur.2017.12.005
157. Kijpaisalratana N, Chutinet A, Travanichakul S, et al. Nonbacterial Thrombotic Endocarditis Related to Adenocarcinoma of the Uterine Cervix. *Case Rep Neurol*. 2020;12(Suppl 1):183-188. Published 2020 Dec 14. doi:10.1159/000507277
158. Muramatsu K, Kawada N, Naganuma H, Ishiwari K, Amagaya S. Recurrent nonbacterial thrombotic endocarditis the day after mitral valve replacement. *J Cardiol Cases*. 2021;25(3):119-122. Published 2021 Aug 26. doi:10.1016/j.jccase.2021.07.014
159. Hadano Y, Ogawa H, Wakeyama T, et al. Nonbacterial thrombotic endocarditis demonstrated by repeat echocardiography. *J Med Ultrason (2001)*. 2013;40(4):453-457. doi:10.1007/s10396-013-0437-4
160. Moţăţăianu A, Maier S, Gothard A, Bajkó Z, Bălaşa R. Severe Fatal Systemic Embolism Due to Non-Bacterial Thrombotic Endocarditis as the Initial Manifestation of Gastric Adenocarcinoma: Case Report. *J Crit Care Med (Targu Mures)*. 2018;4(2):68-73. Published 2018 Apr 1. doi:10.2478/jccm-2018-0008
161. Piovanelli B, Rovetta R, Bonadei I, Vizzardi E, D'Aloia A, Metra M. Nonbacterial thrombotic endocarditis in pancreatic cancer. *Monaldi Arch Chest Dis*. 2013;80(4):189-192. doi:10.4081/monaldi.2013.5236
162. Albright BB, Black JD, Vilardo N, Schwartz PE. Correction of coagulopathy associated with non-bacterial thrombotic endocarditis (NBTE) by surgical debulking in a case of ovarian clear cell carcinoma. *Gynecol Oncol Rep*. 2016;17:13-15. Published 2016 May 7. doi:10.1016/j.gore.2016.05.001
163. Zakka K, Zakka P, Davarpanah A, et al. Nonbacterial Thrombotic Endocarditis and Widespread Skin Necrosis in Newly Diagnosed Lung Adenocarcinoma. *Case Rep Oncol*. 2020;13(1):239-244. Published 2020 Mar 19. doi:10.1159/000506453
164. Kato T, Takama N, Harada T, et al. Nonbacterial Thrombotic Endocarditis-A Rare Case of Acute Libman-Sacks Endocarditis Complicated by Multiple Cerebral Infarcts: Case Report and Literature Review. *CASE (Phila)*. 2020;4(6):507-511. Published 2020 Oct 18. doi:10.1016/j.case.2020.09.005
165. Murtaza G, Iskandar J, Humphrey T, Adhikari S, Kuruvilla A. Lupus-Negative Libman-Sacks Endocarditis Complicated by Catastrophic Antiphospholipid Syndrome. *Cardiol Res*. 2017;8(2):57-62. doi:10.14740/cr534e
166. Wigger O, Windecker S, Bloechlinger S. Nonbacterial thrombotic endocarditis presenting as intracerebral hemorrhage. *Wien Klin Wochenschr*. 2016;128(23-24):922-924. doi:10.1007/s00508-016-1020-y
167. Glass JP. The diagnosis and treatment of stroke in a patient with cancer: nonbacterial thrombotic endocarditis (NBTE): a case report and review. *Clin Neurol Neurosurg*. 1993;95(4):315-318. doi:10.1016/0303-8467(93)90108-s
168. Starobinska E, Robinson EA, Brucks E, Scott S. Marantic endocarditis: incidental infarcts leading to diagnosis of pancreatic cancer. *BMJ Case Rep*. 2018;2018:bcr2018224529. Published 2018 Jun 12. doi:10.1136/bcr-2018-224529
169. Ali N, Konstantinov I, Heath JA, Bhagwat K, Cheung M. Nonbacterial thrombotic endocarditis in a child with non-Hodgkin's lymphoma. *Pediatr Cardiol*. 2012;33(5):843-845. doi:10.1007/s00246-012-0230-y
170. Shatila W, Rizkallah A, Aldin ES, Tfayli A. Nonbacterial thrombotic endocarditis as the sole manifestation of stage IV gastric cancer: a case report. *J Med Case Rep*. 2014;8:267. Published 2014 Aug 4. doi:10.1186/1752-1947-8-267
171. Soeda S, Mathuda N, Hashimoto Y, Yamada H, Fujimori K. Non-bacterial thrombotic endocarditis with systemic embolic events caused by adenomyosis. *J Obstet Gynaecol Res*. 2011;37(12):1838-1841. doi:10.1111/j.1447-0756.2011.01617.x
172. Muhammadzai HZU, Shavadia J, Okpalauwaekwe U, Haddad H. Antemortem diagnosis of nonbacterial thrombotic endocarditis in a patient with previously resected pancreatic adenocarcinoma: a case report. *Eur Heart J Case Rep*. 2020;4(5):1-4. Published 2020 Aug 30. doi:10.1093/ehjcr/ytaa167
173. Oueida Z, Scola M. Ovarian Clear Cell Carcinoma Presenting as Non-bacterial Thrombotic Endocarditis and Systemic Embolization. *World J Oncol*. 2011;2(5):270-274. doi:10.4021/wjon367e
174. Singh S, Dass A, Jain S, Varma S, Bannerjee AK, Sharma BK. Fatal non-bacterial thrombotic endocarditis following viperine bite. *Intern Med*. 1998;37(3):342-344. doi:10.2169/internalmedicine.37.342
175. Tanaka H, Ito M, Yoshida K, Asakura T, Taniguchi H. Nonbacterial thrombotic endocarditis complicated with stage Ia ovarian cancer. *Int J Clin Oncol*. 2009;14(4):369-371. doi:10.1007/s10147-008-0852-5
176. Tanawuttiwat T, Dia M, Hanif T, Mihailescu M. Double-valve Libman-Sacks endocarditis causing ventricular fibrillation cardiac arrest. *Tex Heart Inst J*. 2011;38(3):295-297.
177. Erturk NK, Erturk A, Basaran D, Ozgul N. Synchronous Ovarian and Endometrial Endometrioid Adenocarcinoma Presenting with Nonbacterial Thrombotic Endocarditis and Pulmonary Thromboembolism: Adenocarcinoma with Thrombotic Events. *Case Rep Obstet Gynecol*. 2015;2015:825404. doi:10.1155/2015/825404
178. Morimoto S, Tanaka J, Saito Y, et al. Non-bacterial thrombotic endocarditis in a Trousseau syndrome patient with stomach cancer: A case report. *Geriatr Gerontol Int*. 2016;16(10):1171-1172. doi:10.1111/ggi.12694
179. Krawczyk M, Budhram A, Sposato LA. Ischemic Stroke From Libman-Sacks Endocarditis Not Associated With Antiphospholipid Antibodies: Good Clinical Outcome Without Anticoagulation. *JACC Case Rep*. 2019;1(3):297-300. Published 2019 Sep 11. doi:10.1016/j.jaccas.2019.06.032
180. Yokoyama S, Iwano H, Yamada S, et al. Long-term echocardiographic evaluation of valvular lesions in a patient with nonbacterial thrombotic endocarditis associated with advanced uterine cancer. *J Cardiol Cases*. 2016;14(3):82-86. Published 2016 Jun 2. doi:10.1016/j.jccase.2016.04.005
181. Lønnebakken MT, Gerdts E. Libman-Sacks endocarditis and cerebral embolization in antiphospholipid syndrome. *Eur J Echocardiogr*. 2008;9(1):192-193. doi:10.1093/ejechocard/jem067
182. Lepour M, Wieërs GJFG, Vereeke J, Wauters A. Reversibility of valve regurgitation due to cancer-related non-bacterial thrombotic endocarditis after switching direct oral anticoagulation for heparin. BMJ Case Rep. 2022;15(3):e247672. Published 2022 Mar 16. doi:10.1136/bcr-2021-247672
183. Perrone F, Biagi A, Facchinetti F, et al. Systemic thromboembolism from a misdiagnosed non-bacterial thrombotic endocarditis in a patient with lung cancer: A case report. *Oncol Lett*. 2020;20(5):194. doi:10.3892/ol.2020.12056
184. Lobo Ferreira T, Alves R, Judas T, Delerue MF. Marantic endocarditis and paraneoplastic pulmonary embolism. *BMJ Case Rep*. 2017;2017:bcr2017220217. Published 2017 Jul 14. doi:10.1136/bcr-2017-220217
185. Mantovani F, Navazio A, Barbieri A, Boriani G. A first described case of cancer-associated non-bacterial thrombotic endocarditis in the era of direct oral anticoagulants. *Thromb Res*. 2017;149:45-47. doi:10.1016/j.thromres.2016.11.016
186. Jameson GS, Ramanathan RK, Borad MJ, Downhour M, Korn R, Von Hoff D. Marantic Endocarditis Associated with Pancreatic Cancer: A Case Series. *Case Rep Gastroenterol*. 2009;3(1):67-71. Published 2009 Apr 15. doi:10.1159/000207195
187. Numnum TM, Leath CA 3rd, Straughn MJ Jr. Synchronous primary endometrial and ovarian carcinoma in a patient with marantic endocarditis. *Obstet Gynecol*. 2006;108(3 Pt 2):748-750. doi:10.1097/01.AOG.0000190220.13074.87
188. Shibata N, Matsumoto K, Kitamura S, Sakashita A, Kizawa Y, Hirata KI. Nonbacterial Thrombotic Endocarditis Concomitant with Repeated Systemic Embolization That Received Palliative Care Based on the Antemortem Diagnosis. *Intern Med*. 2018;57(24):3559-3563. doi:10.2169/internalmedicine.1381-18
189. Murata A, Kagiyama N, Miyazaki S, et al. Severe Aortic Regurgitation by Nonbacterial Thrombotic Endocarditis Treated with Anticoagulation Therapy. *CASE (Phila)*. 2022;6(5):205-208. Published 2022 May 20. doi:10.1016/j.case.2022.04.002
190. Schlittler LA, Dallagasperina VW, Schavinski C, Baggio AP, Lazaretti NS, Villaroel RU. Marantic endocarditis and adenocarcinoma of unknown primary site. *Arq Bras Cardiol*. 2011;96(4):e73-e75.
191. Tei T, Nomura T, Naito D, et al. Effective surgical treatment for controlling the acute heart failure induced by acutely progressed mitral regurgitation with nonbacterial thrombotic endocarditis. *J Cardiol Cases*. 2010;2(2):e59-e62. Published 2010 Mar 24. doi:10.1016/j.jccase.2010.03.001
192. Takeshita S, Ogata T, Mera H, et al. Multiple Thrombi in the Heart in Trousseau Syndrome Caused by Pancreatic Carcinoma. *J Stroke Cerebrovasc Dis*. 2018;27(5):e75-e77. doi:10.1016/j.jstrokecerebrovasdis.2017.12.005
193. Ferlan G, Fiorella A, De Pasquale C, Tunzi F. Primary coronary embolism as an unusual manifestation of nonbacterial thrombotic endocarditis in a patient with gastric cancer. *Cardiol Res Pract*. 2010;2010:319732. doi:10.4061/2010/319732
194. Hofstra JH, Timmer JR, Breeman A, Havenith MG. Non-bacterial thrombotic endocarditis in metastatic caecal adenocarcinoma. *Neth Heart J*. 2009;17(9):349-350. doi:10.1007/BF03086282
195. Fox IS, Spence AM, Wheelis RF, Healey LA. Cerebral embolism in Libman-Sacks endocarditis. *Neurology*. 1980;30(5):487-491. doi:10.1212/wnl.30.5.487
196. Malvar B, Almeida FM, Rebocho L, Moniz JC, Azevedo F. Cerebral embolism from Libman-Sacks endocarditis. *BMJ Case Rep*. 2011;2011:bcr0420114071. Published 2011 Jun 30. doi:10.1136/bcr.04.2011.4071
197. Kang AH, Graves CR. Libman-Sacks endocarditis in a pregnant woman with acute respiratory distress syndrome. *Obstet Gynecol*. 1999;93(5 Pt 2):819-821. doi:10.1016/s0029-7844(98)00458-x
198. Shen HC, Hsu YF, Chiang CL. Successful Treatment of Nonbacterial Thrombotic Endocarditis and Disseminated Intravascular Coagulation in a Patient With Advanced Lung Adenocarcinoma Using Osimertinib. *JTO Clin Res Rep*. 2020;1(3):100066. Published 2020 Jun 1. doi:10.1016/j.jtocrr.2020.100066
199. Liang H, Ma C, Chen X. Case report: Mitral valve replacement for Libman-Sacks endocarditis and cerebral embolism of primary antiphospholipid syndrome. *Front Cardiovasc Med*. 2022;9:985111. Published 2022 Aug 18. doi:10.3389/fcvm.2022.985111
200. Iranpour A, Mahmoodian R, Haghighi A, et al. A 34 year old man with purple discoloration and paresthesia. *Int J Hematol Oncol Stem Cell Res*. 2014;8(1):41-45.
201. Norisada K, Tanaka H, Onishi T, et al. Nonbacterial thrombotic endocarditis associated with cancer of unknown origin complicated with thrombus in the left auricular appendage: case report. *Cardiovasc Ultrasound*. 2011;9:8. Published 2011 Feb 28. doi:10.1186/1476-7120-9-8
202. Dichtwald S, Meyer A, Ifrach N. Catastrophic anti-phospholipid syndrome with Libman-Sacks endocarditis following eltrombopag therapy for immune thrombocytopenic purpura: A case report. *Lupus*. 2021;30(14):2304-2309. doi:10.1177/09612033211065140
203. Farooqui AA, Ashraf R, D'Ornellas R, Aslam A, Marcelin M, Shetty V. Tricuspid Valve Vegetation Secondary to Ovarian Cancer Leading to Acute Stroke via Pulmonary Arteriovenous Malformation. *Cureus*. 2021;13(8):e17136. Published 2021 Aug 12. doi:10.7759/cureus.17136
204. Lee JM, Lim JH, Kim JS, et al. Multiple hypercoagulability disorders at presentation of non-small-cell lung cancer. *Tuberc Respir Dis (Seoul)*. 2014;77(1):34-37. doi:10.4046/trd.2014.77.1.34
205. Suzuki A, Asazuma N, Kikuchi E, et al. "Possible primary antiphospholipid syndrome" with concurrent diffuse alveolar hemorrhaging and Libman-Sacks endocarditis mimicking catastrophic antiphospholipid syndrome. *Intern Med*. 2012;51(7):813-816. doi:10.2169/internalmedicine.51.6592
206. Arnautovic JZ, Yamasaki H, Rosman HS. Multiple embolic strokes as a result of Libman-Sacks endocarditis associated with lupus and secondary antiphospholipid antibody syndrome: a case report. *Eur Heart J Case Rep*. 2018;2(3):yty094. Published 2018 Aug 17. doi:10.1093/ehjcr/yty094
207. Miller ES, Hussein HM, Gertner E. A 56-Year-Old Woman with Recurrent Strokes: A Clear Case with a Therapeutic Dilemma. *Case Rep Neurol*. 2021;13(1):100-107. Published 2021 Feb 15. doi:10.1159/000512126
208. Thomas LM, Shaikh NA, Pradeep R. An extraordinary case of recurrent stroke, disseminated thrombosis and endocarditis. *BMJ Case Rep*. 2018;2018:bcr2018224172. Published 2018 Jun 4. doi:10.1136/bcr-2018-224172
209. Khan RM, Namas R, Parikh S, Rubin B. Embolic Stroke as the Initial Manifestation of Systemic Lupus Erythematosus. *Case Rep Rheumatol*. 2015;2015:373201. doi:10.1155/2015/373201
210. Patnaik, S, Fernandez, G, Aliling, J. et al. NON-BACTERIAL THROMBOTIC ENDOCARDITIS IN A PATIENT WITH PRIMARY ANTIPHOSPHOLIPID SYNDROME. JACC. 2016 Apr, 67 (13_Supplement) 1110. <https://doi.org/10.1016/S0735-1097(16)31111-1>
211. Sánchez-Enrique C, Vilacosta I, Moreno HG, et al. Infected marantic endocarditis with leukemoid reaction. *Circ J*. 2014;78(9):2325-2327. doi:10.1253/circj.cj-14-0079
212. Hoofer WD. Hypercoagulability and verrucous endocarditis associated with adenocarcinoma of the lung. *Ann Thorac Surg*. 1968;6(2):181-184. doi:10.1016/s0003-4975(10)66011-0
213. Khachatoorian, Y, Lasam, G, Sabharwal, B. et al. LIBMAN-SACKS ENDOCARDITIS TREATMENT WITH WARFARIN AFTER RIVAROXABAN FAILURE: BREAKING THE CASCADE OF DELETERIOUS SEQUELAE. JACC. 2022 Mar, 79 (9_Supplement) 3434 <https://doi.org/10.1016/S0735-1097(22)04425-4>
214. González A, Vergara M, Heredia A, Llanos L, Araos F. Accidente cerebrovascular embólico por endocarditis de Libman-Sacks como manifestación inicial de síndrome antifosfolípidos primario en una paciente de 17 años [Libman-Sacks endocarditis associated with antiphospholipid syndrome. Report of one case]. *Rev Med Chil*. 2013;141(9):1206-1210. doi:10.4067/S0034-98872013000900015
215. de la Iglesia Fanjul I, Alonso Rodríguez D. Endocarditis trombótica no bacteriana y neoplasia pulmonar [Non-bacterial thrombotic endocarditis and lung neoplasm]. *An Med Interna*. 2006;23(10):487-489.
216. [SALDARRIAGA, Carolina](http://www.scielo.org.co/cgi-bin/wxis.exe/iah/?IsisScript=iah/iah.xis&base=article%5Edlibrary&format=iso.pft&lang=i&nextAction=lnk&indexSearch=AU&exprSearch=SALDARRIAGA,+CAROLINA); [MUNERA, Ana G.](http://www.scielo.org.co/cgi-bin/wxis.exe/iah/?IsisScript=iah/iah.xis&base=article%5Edlibrary&format=iso.pft&lang=i&nextAction=lnk&indexSearch=AU&exprSearch=MUNERA,+ANA+G.)  and  [DUQUE, Mauricio](http://www.scielo.org.co/cgi-bin/wxis.exe/iah/?IsisScript=iah/iah.xis&base=article%5Edlibrary&format=iso.pft&lang=i&nextAction=lnk&indexSearch=AU&exprSearch=DUQUE,+MAURICIO). **Libman-Sacks endocarditis.** *Rev. Colomb. Cardiol.* [online]. 2015, vol.22, n.3, pp.144-148. ISSN 0120-5633.  <https://doi.org/10.1016/j.rccar.2015.03.010>.
217. Bathina JD, Daher IN, Plana JC, Durand JB, Yusuf SW. Acute myocardial infarction associated with nonbacterial thrombotic endocarditis. *Tex Heart Inst J*. 2010;37(2):208-212.
218. Mohammadi Kebar et al., Libman-Sacks endocarditis in SLE with APS
