# Supplementary material for: Stroke in non-bacterial thrombotic endocarditis: a patient-level systematic review of clinical characteristics, management, and outcomes
Source: Front Stroke. 2026 Aug 14;5:1915602. doi: 10.3389/fstro.2026.1915602 (PMC13521837; doi:10.3389/fstro.2026.1915602)
Supplement: Supplementary file 2 [file Table_1.docx]

Supplementary Table 1. Methodological assessment of case reports and case series for risk of bias

| Domain | Explanatory questions |
| --- | --- |
| Selection | 1. Does the patient(s) represent(s) the whole experience of the investigator (center) or is the selection method unclear to the extent that other patients with similar presentations may not have been reported? |
| Ascertainment | 2. Was the exposure adequately ascertained? 3. Was the outcome adequately ascertained? |
| Causality | 4. Were other alternative causes that may explain the observation ruled out? 5. Was there a challenge/rechallenge phenomenon? ^**^ 6. Was there a dose–response effect? ^*^ 7. Was follow-up long enough for outcomes to occur? |
| Reporting | 8. Is the case(s) described with sufficient details to allow other investigators to replicate the research or to allow practitioners to make inferences related to their own practice? |

^**^ Questions 5 and 6 were not included in the quality assessment of included case reports/series as they were not relevant to this review and they are proposed to investigate cases of adverse drug events as described by Murad et al.
